# Loss of* SLC25A20* in Pancreatic Adenocarcinoma Reversed the Tumor-Promoting Effects of a High-Fat Diet

**DOI:** 10.7150/thno.114912

**Published:** 2025-05-25

**Authors:** Sang Myung Woo, Ho Lee, Joon Hee Kang, Mingyu Kang, Wonyoung Choi, Sung Hoon Sim, Jung Won Chun, Nayoung Han, Kyung-Hee Kim, Woojin Ham, Woosol Hong, Chaeyoung Kim, Jeong Hwan Park, Dawool Han, Jong In Yook, Woo Jin Lee, Soo-Youl Kim

**Affiliations:** 1 Research Institute, National Cancer Center, Goyang, Republic of Korea; 2 Department of Cancer Biomedical Science, National Cancer Center Graduate School of Cancer Science and Policy, Goyang, Republic of Korea; 3 Center for Liver and Pancreatobiliary Cancer, National Cancer Center, Goyang, Republic of Korea.; 4 Cancer Molecular Biology Branch, Research Institute, National Cancer Center, Goyang, Republic of Korea; 5 New Cancer Cure-Bio Co., Goyang, Republic of Korea; 6 Therapeutic Resistance Research Branch, Research Institute and Hospital, National Cancer Center, Goyang, Republic of Korea; 7 Department of Pathology, Research Institute and Hospital, National Cancer Center, Goyang, Republic of Korea; 8 Proteomics Core Facility, Research Core Center, Research Institute, National Cancer Center, Goyang, Republic of Korea; 9 Department of Oral Pathology, Yonsei University College of Dentistry, Seoul, Korea

**Keywords:** PDAC, High-fat diet, Fatty acid oxidation, Pancreatic cancer, SLC25A20

## Abstract

**Rationale:** Although it is known that High-fat diet (HFD) promotes the development of pancreatic ductal adenocarcinoma (PDAC), no direct link between HFD and cancer has been identified. Previously, we showed that ATP production by cancer cells depends on fatty acid oxidation (FAO); therefore, we hypothesized that blocking FAO may prevent HFD-induced promotion of PDAC growth.

**Methods:** To determine whether FAO is increased in PDAC patients, we analyzed a tissue microarray by immunohistochemical staining to detect carnitine palmitoyl transferase I. To block FAO, *SLC25A20* (carnitine-acylcarnitine carrier) was knocked down in cancer cells, which was implanted for xenograft in mice and treated with a high-fat diet (HFD, 60% fat). To compare cancer development including survival rates, and histopathological differences were analyzed by crossbreeding of KPC mice (*Kras^G12D/+;^ Trp53^R172H/+;^ Pdx1-Cre*) with KPC/*Slc25a20^+/-^* mice.

**Results:** *SLC25A20* knockdown in cancer cells reduced ATP production and inhibited cell growth. Proteome analysis revealed that *SLC25A20* knockdown reduced cancer cell growth significantly due to inactivation of mTOR via decreased ATP production, ultimately leading to cell death. The median survival time of KPC/*Slc25a20^+/-^* tumor-bearing mice was 3.1 weeks longer than that of KPC tumor-bearing mice. In mice fed an HFD, the growth of xenografts derived from *SLC25A20* knockdown PDAC cells was 65-95% lower than that of xenografts derived from control cells.

**Conclusion:** Blocking FAO by *SLC25A20* knockdown reversed HFD-induced promotion of PDAC growth.

## Introduction

Studies have shown that high-fat diet (HFD) affects tumor metabolism, which can promote tumor growth and development in various cancers such as endometrial cancer 1, prostate cancer 2, and colorectal cancer 3. Although the exact mechanism varies by cancer site, including PDAC, the precise mechanisms underlying these associations between HFD and tumor promotion are still being explored and not fully understood 1-4. Obesity is a very complex disease, with genetic, environmental, and multifactorial causes, so it is very difficult to establish a link between obesity and cancer with a general mechanism. Therefore, we investigated the link between HFD-induced obesity and tumor promotion using mice models. Leptin-deficient *ob/ob* mice are obese mice that are suitable models for investigating the indirect causes of cancer and the development process of cancer 5. Balb/c to high-fat diet (HFD) model of tumor promotion is a good model to investigate tumor promotion directly by obesity signature of the increased BMI 6. HFD is not only associated with an increased risk of developing cancer 7, but it can also increase the risk of cancer recurrence and mortality 8. Three significant mechanisms linking to cancer have been proposed to explain how HFD-induced obesity increases the risk of cancer 9. First, adipose tissue with HFD secretes vast amounts of estrogen, which is linked to an increased risk of breast, ovary, and other types of cancer 10. Second, an increased body mass index (BMI) causes hyperinsulinemia (elevated serum levels of insulin) and increased levels of insulin-like growth factor-1 (IGF-1) 11. The level of IGF-1 in cancer patients is often higher than that in those without cancer, and levels correlate with an increased risk of colon, renal, prostate, and endometrial cancer 11, 12. Third, adipokines such as leptin (which have proinflammatory effects) secreted by adipose tissue may promote cancer development 13. Increasing levels of leptin correlate with increased body fat percentage, which in turn increases the risk of biliary tract and other types of cancer 14. However, we still do not understand how HFD-induced obesity drives cancer development directly.

Animals fed a HFD are used as models for obesity, with data being compared with those obtained from animals fed a calorie-balanced no-fat diet (NFD). The diet-induced obesity model is an animal model based on high-fat or high-density diets 15. Previously, we used an HFD mouse model and a homograft KC (*Kras^G12D/+;^ Pdx1-Cre*) model to show that a calorie-balanced HFD results in a significant increase in the growth of human PDAC xenograft tumors 16. Under normal proliferative conditions, fatty acid oxidation (FAO) is a major source of ATP production in cancer cells and operates independently of obesity 17. FAO is responsible for the production of key energy metabolites such as NADH and ATP, as demonstrated by a series of experiments in which glucose-free culture conditions did not alter ATP levels in cancer cells 16-18. Therefore, we propose that HFD-induced tumor promotion can be explained directly by overproduction of ATP via FAO. The present study asks the following questions: is FAO increased in pancreatic cancer? Does HFD promote the growth of pancreatic cancer? And does knocking down FAO-related genes in pancreatic cancer cells reverse HFD-induced tumor growth?

## Results

### Increased expression of FAO is associated with a significant reduction in the survival of PDAC

**Figure 1:**
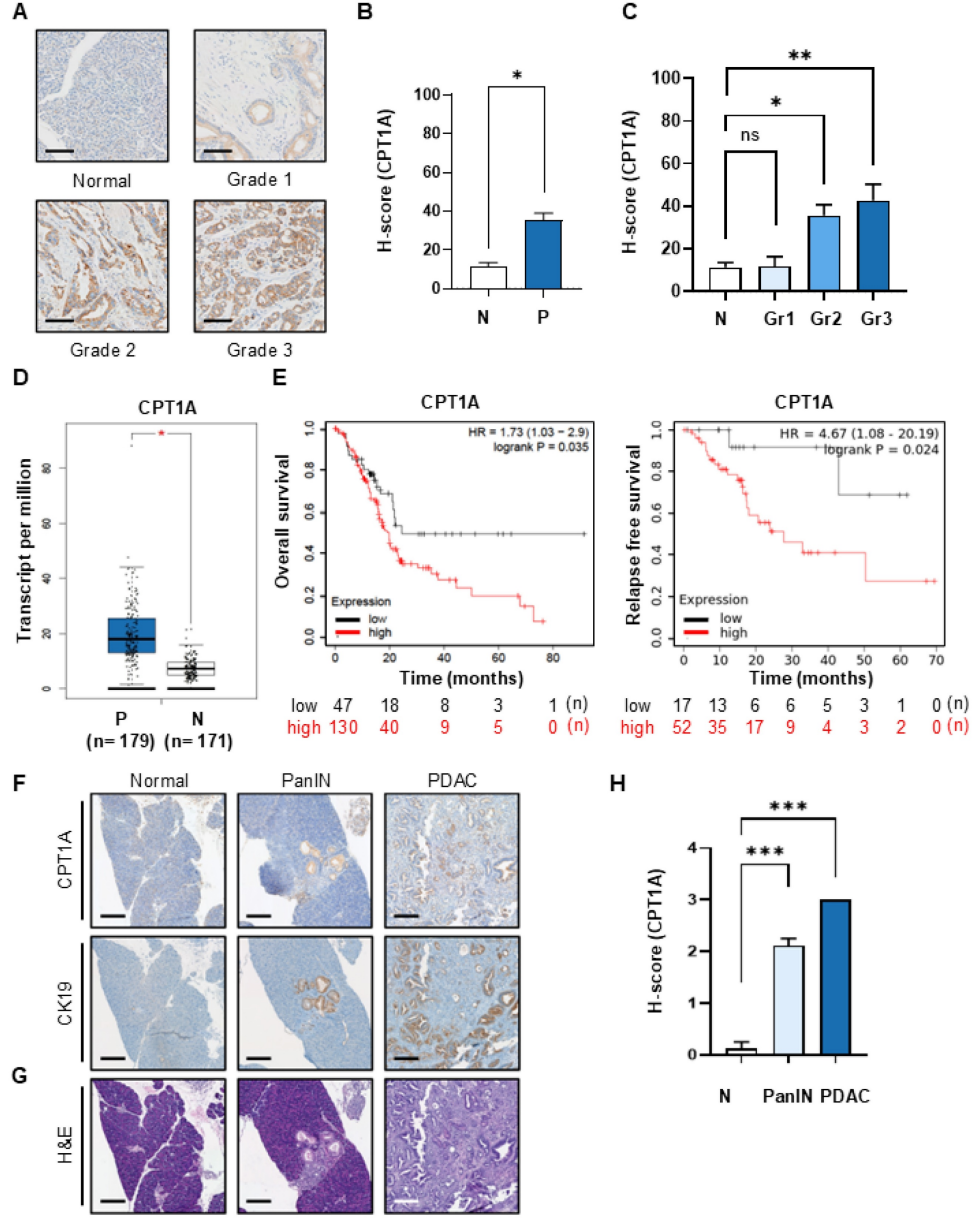
** Expression of the FAO marker CPT1A increases as the grade of PDAC increases. (A)** Immunohistochemical (IHC) staining of CPT1A using tissue microarrays derived from normal (n = 27) controls and patients with grade 1 (n = 18), grade 2 (n = 74), or grade 3 (n = 45) PDAC. Representative images were selected from the TMA (Figure S1A). Scale bar = 100 µm. **(B)** H-scores for normal (N, n = 27) and tumor tissues (P, n = 137), were calculated using Inform software. The median H-score for CPT1A in normal and pancreatic cancer tissues was 11.30 and 35.50, respectively. **(C)** H-Scores for normal (N, n = 27), grade 1 (Gr1, n = 18), grade 2 (Gr2, n = 74), and grade 3 (Gr3, n = 45) pancreatic tissue. The median H-score for CPT1A in grade 1, 2, and 3 PDAC tissues was 11.78, 35.64, and 42.71, respectively. **(D)** CPT1A mRNA levels in PDAC patients (P, n = 179) were compared with those in matched normal (N, n = 171) controls using the GEPIA website (http://gepia.cancer-pku.cn/). **(E)** Pancreatic adenocarcinoma datasets related to CPT1A expression were analyzed using the Kaplan-Meier Plotter (https://kmplot.com/analysis/). PDAC patients were shown with high expression of CPT1A (red line) and with low expression of CPT1A (black line). **(F, G)**. Representative IHC images of CPT1A and CK-19 expression (F), and H&E (G) staining of the pancreas from normal, low-grade PanIN, high-grade PanIN, and PDAC KPC mice (*Kras^G12D^; Trp53^R172H^; Pdx1-Cre*). **(H)**. H-score scores for normal (n = 8), PanIN (n = 27), and PDAC (n = 5) tissues. Scale bar = 150 µm. The median of CPT1A H-scores in normal, PanIN, and PDAC tissues were 0.125, 2.111, and 3, respectively. *p < 0.05, **p < 0.01, ***p < 0.001.

To investigate the impact of FAO on the development of pancreatic cancer, we compared the expression of CPT1A, a key marker of FAO, by performing immunohistochemical analysis of tissues from patients with pancreatic cancer (Figure 1A and Figure S1A). CPT1A is essential for b-oxidation of long-chain fatty acids. This transfer system catalyzes the synthesis of acyl-carnitine, which is necessary for FAO. The average H-score for CPT1A in normal tissue was 11.3, while that in pancreatic cancer tissue was an average of 35.5, i.e., a 3.14-fold increase in expression compared with that in normal tissue (Figure 1B). When assessed against grade, the H-score values for pancreatic cancer tissues were as follows: 11.8 for grade 1, 35.6 for grade 2, and 42.7 for grade 3 (Figure 1C). The increase in CPT1A expressions observed in grade 2 and grade 3 pancreatic cancer was statistically significant; indeed, there was an up to 4-fold increase in the H-score between grade 3 cancer and normal tissue. Expression of CPT1A mRNA also increased in patients with pancreatic cancer, as shown by the TCGA data (Figure 1D). There was an inverse correlation between high CPT1A expression and OS and RFS (Figure 1E). Next, we established a spontaneous pancreatic cancer KPC mouse model (*Kras^G12D/+;^ Trp53^R172H/+;^ Pdx1-Cre*) and validated the increase in CPT1A expression during cancer progression. Histological analysis of pancreatic tissue from KPC mice was performed by H&E staining to identify normal, PanIN, and PDAC lesions. We then compared CPT1A expression levels between the selected tissues (Figure 1F and Figure S1B). Low CPT1A expression was observed in normal mouse pancreatic tissue; however, PanIN, and PDAC tissues showed higher expression of CPT1A than normal tissue. This increase of CPT1A expression correlated with the increase of CK-19 (Figure 1F & G, Figure S1C), which showed statistically significant (Figure 1H).

To evaluate the impact of HFD on cancer growth and development in the KPC mouse model, we fed them either an HFD (60% fat) or an RD (regular diet, 18% fat). The diets were initiated at 12 weeks of age, which is when pancreatic cancer typically develops in this model. Mice in the HFD group exhibited an average weight increase of 24% when compared with the RD group (Figure S2A). Additionally, the average BMI values for the HFD and RD groups were 0.43 and 0.36, respectively (Figure S2B). The median survival time of KPC mice in the HFD group (16 weeks) was 2 weeks shorter than that of mice in the RD group (18 weeks; p = 0.003; Figure S2C). Immunohistochemical staining using IHC (CK19) and H&E staining to identify PanIN stage lesions with similar histological features between groups revealed a 2.2-fold increase in the expression of the FAO marker CPT1A in the HFD group compared to the RD group (Figure S2D, Figure S3A & B).

### An increase in fatty acid supplementation promotes PDAC growth directly *in vitro*

**Figure 2:**
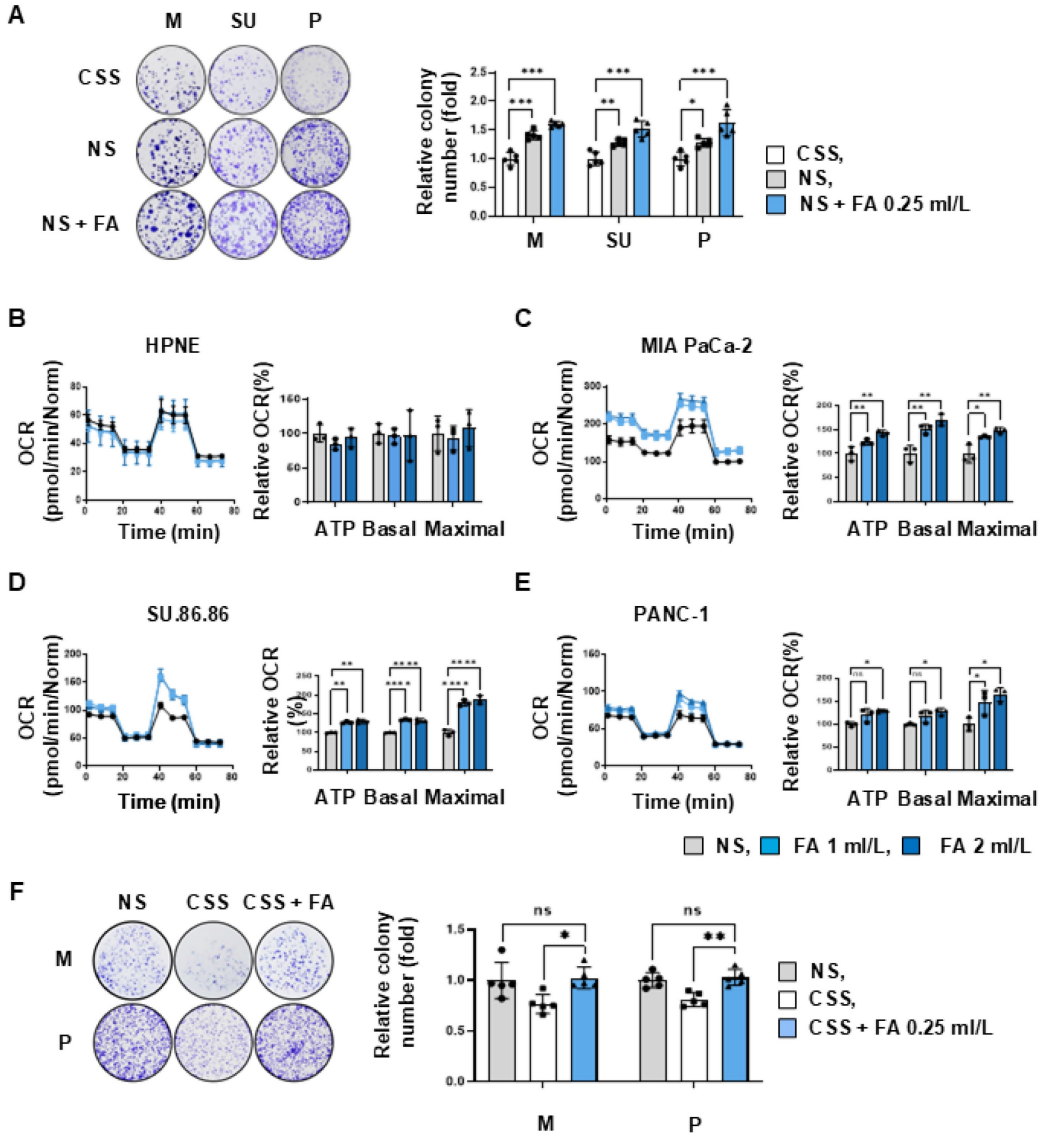
** Cancer cells rely on fatty acids to produce ATP and promote growth. (A)** To test whether growth of pancreatic cancer cells was evaluated in a clonogenic assay after culture for 2 weeks in the presence of charcoal-stripped serum (CSS), or normal serum (NS, 10%), or NS medium supplemented with fatty acids (NS + FA 0.25 ml/L) (n=5). **(B-E)** The increase in ATP production following FA dose-dependent treatment was measured using a seahorse XFe96 analyzer. (B) In hTERT-HPNE normal cells, additional treatment with FA did not cause changes in ATP synthesis. ATP levels were FA dose-dependently increased in MIA PaCa-2 (C), SU.86.86 (D), and PANC-1 (E). **(F)** To test whether the reduced growth of pancreatic cancer cells by CSS treatment (A) can be rescued by fatty acid treatment (CSS + FA 0.25 ml/L) (n=5). Data was shown as the mean ± SD of at least three experiments. *p < 0.05, **p < 0.01, ***p < 0.001.

To investigate the effects of fatty acids on pancreatic cancer cells, we cultured representative pancreatic cancer cell lines MIA PaCa-2, SU.86.86, and PANC-1 in a medium supplemented with either normal serum (NS) or charcoal-stripped serum (CSS), which has a 99% reduction in free fatty acid content 19. Pancreatic cancer cells cultured in CSS medium showed an approximately 28-41% reduction in colony formation compared to the control (NS medium, Figure 2A). When the NS medium was supplemented with fatty acids (0.25 ml/L, NS+FA), colony formation by all cell lines increased by about 22% compared with that observed in the NS medium alone (Figure 2A). Therefore, the increase of colony formation between CSS and NS + FA was about 43-63%. This implies that PDAC cancer cell lines depend on fatty acids to drive proliferation. To determine whether growth promotion by fatty acids is related to ATP production, we conducted oxygen consumption rate (OCR) assays after treating cells with fatty acids at varying concentrations. In normal pancreatic cells (hTERT-HPNE), an increase in fatty acid uptake did not affect intracellular basal respiration or ATP production (Figure 2B). By contrast, treatment of the pancreatic cancer cell line MIA PaCa-2 with fatty acids (2 ml/L) increased basal respiration by 71%, and ATP production by 43%, when compared with NS (Figure 2C). In SU.86.86 cells, fatty acid treatment increased basal respiration by 32 % and ATP production by 30% (Figure 2D). Fatty acid treatment of PANC-1 cells increased basal respiration by 27% and ATP production by 29% (Figure 2E), To demonstrate that fatty acid depletion was the main cause of the inhibition of cancer cell proliferation by CSS, we added fatty acids (0.25 ml/L, CSS+FA) to the CSS medium to test whether cancer cell proliferation was restored (Figure 2F). We observed that FA supplement after CSS treatment restored about 70% or more of the CSS-induced reduction in colony growth (Figure 2F).

### Blocking FAO by knocking down SLC25A20 suppresses ATP production

**Figure 3:**
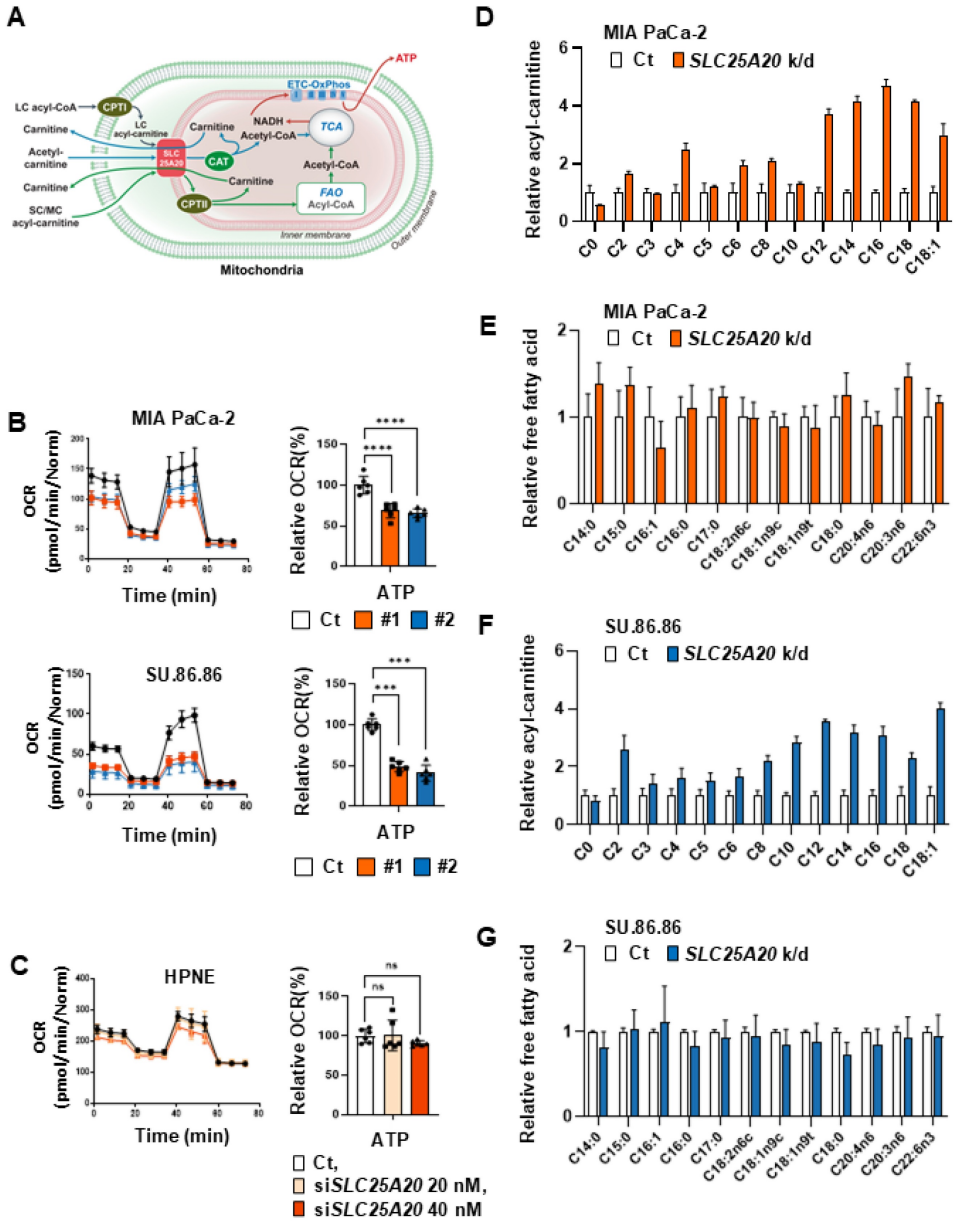
***SLC25A20* knockdown reduces ATP production in PDAC cells. (A)** Summary of fatty acid transportation into mitochondria for FAO. CPTI/II (carnitine palmitoyl transferase I/II), CAT (carnitine acetyltransferase), ETC-OxPhos (electron transfer complex-oxidative phosphorylation). **(B)** Immunoblot analysis was shown in Figure S4A. ATP levels in PDAC cell lines MIA PaCa-2 and SU.86.86 were then measured by Seahorse XFe96 analysis. All data were normalized by SRB analysis. **(C)** Immunoblot analysis was shown in Figure S4B. ATP levels in hTERT-HPNE cell line were measured with Seahorse XFe96. All data were normalized by measuring SRB assay. **(D, E)** The metabolomes of various acyl-carnitines (D) and fatty acids (E) in MIA PaCa-2 cells without (control) and with *SLC25A20* knockdown (orange) were analyzed by MS/MS. The y-axis shows the results of the *SLC25A20* knockdown, expressed as a relative ratio with the control set to 1.0. **(F and G)** The metabolomes of various acyl-carnitines (F) and fatty acids (G) in SU.86.86 cells without (control) and with *SLC25A20* knockdown (blue) were analyzed by MS/MS. The y-axis shows the results of the *SLC25A20* knockdown, expressed as a relative fold ratio, with the control set to 1.0. Data are expressed as the mean ± SD of three independent experiments. *p < 0.05, **p < 0.01, ***p < 0.001.

Intracellular ATP production via FAO occurs predominantly in the mitochondria (Figure 3A). To briefly summarize FAO in the mitochondria: long-chain fatty acids can be activated as a long-chain (LC) acyl-CoA; however, they cannot freely diffuse through the mitochondrial inner membrane. Therefore, they require a shuttle system for transport to the mitochondrial matrix (Figure 3A) 20. SLC25A20 catalyzes the esterification of hydrophobic carbon chains C2 (acetyl)-C16 (palmitoyl) to the hydroxyl group of carnitine 21. For this reason, it is thought that blocking the SLC25A20 will effectively disrupt the transport of the various substrates required for the FAO reaction.

To determine whether inhibiting components involved in FAO reduce intracellular production of ATP, we knocked down *SLC25A20* and then conducted OCR assays. The results revealed that shRNA-mediated knockdown of *SLC25A20* in the pancreatic cancer cell line MIA PaCa-2 reduced basal respiration by 25-40% and ATP production by 34-43% (Figure 3B, Figure S4A & C). ShRNA-mediated knockdown of *SLC25A20* in the pancreatic cancer cell lines SU.86.86 reduced ATP production by 16-60% (Figure 3B, Figure S4A & C); however, there was no change in ATP production in pancreatic normal ductal epithelial cells (hTERT-HPNE) after *SLC25A20* knockdown (Figure 3C, Figure S4B & D). This implies that hTERT-HPNE did not utilize FAO to generate ATP (Figure 3C). *SLC25A20* knockdown showed that the accumulation of acyl-carnitines in cancer cells (Figure 3D). However, when *SLC25A20* was knocked down with siRNA in PDAC cells, ATP dropped by about 40% (Figure 3B and Figure S4E). We knocked down *SLC25A20* in cancer cells and added glutamine to test whether the decreased ATP levels were restored (Figure S4F). Therefore, after *SLC25A20* knockdown for 48 hours, we added 2.5 mM and 5 mM glutamine in the culture media and observed whether ATP levels were restored after 24 hours (Figure S4F). In the MIA PaCa-2 and Su.86.86 cell lines, *SLC25A20* knockdown reduced ATP levels by 35% and 50%, respectively, and glutamine treatment with 2.5 and 5.0 mM did not affect ATP levels at all (Figure S4F). To test whether FAO knockdown induces this pathway, SLC25A20 was knocked down for 48 hours and lactate production was measured (Figure S4G). The experimental results showed that there was no change in lactate production in both MIA PaCa-3 and SU.86.86 cell lines. The same was true for other cancer cell types, including glioblastoma, breast cancer, lung cancer, colon cancer, and liver cancer, where* SLC25A20* knockdown reduced ATP production by 26-70% at a 40 nM siRNA concentration (Figure S4H & I). *SLC25A20* knockdown in MIA PaCa-2 cells increased the levels of long-chain acyl-carnitines such as C18:1-carnitine, C18-carnitine, C16-carnitine, and C-12-carnitine by 2-3 fold (Figure 3D). In addition, mid-chain acyl-carnitines such as C6-carnitine, and C8-carnitine also increased by more than twofold. This increase appears to reduce the amount of free carnitine C0 that is not recovered (Figure 3D).* SLC25A20* knockdown in SU.86.86 cells increased the levels of long-chain acyl-carnitines such as C18-carnitine, C16-carnitine, C14-carnitine, and C-12-carnitine by 2-3 fold (Figure 3F). In addition, short-chain or mid-chain acyl-carnitines such as C2-carnitine, C8-carnitine, and C10-carnitine also increased by more than twofold. This increase appears to reduce the amount of free carnitine C0 in both cell lines that was not recovered from SLC25A20 shuttle (Figure 3F). By contrast, free fatty acid levels did not change in MIA PaCa-2 or SU.86.86 cells following *SLC25A20* knockdown (Figure 3E & G). *CPT1A* knockdown in pancreatic cancer cell lines MIA PaCa-2 and SU.86.86 reduced ATP production by 40-50% (Figure S5A & B). To analyze the changes in intracellular acyl-carnitine and free fatty acid levels induced by *SLC25A20* knockdown, we conducted LC-MS/MS analysis.

### Mitochondrial inactivation through SLC25A20 knockdown slows PDAC growth significantly

**Figure 4:**
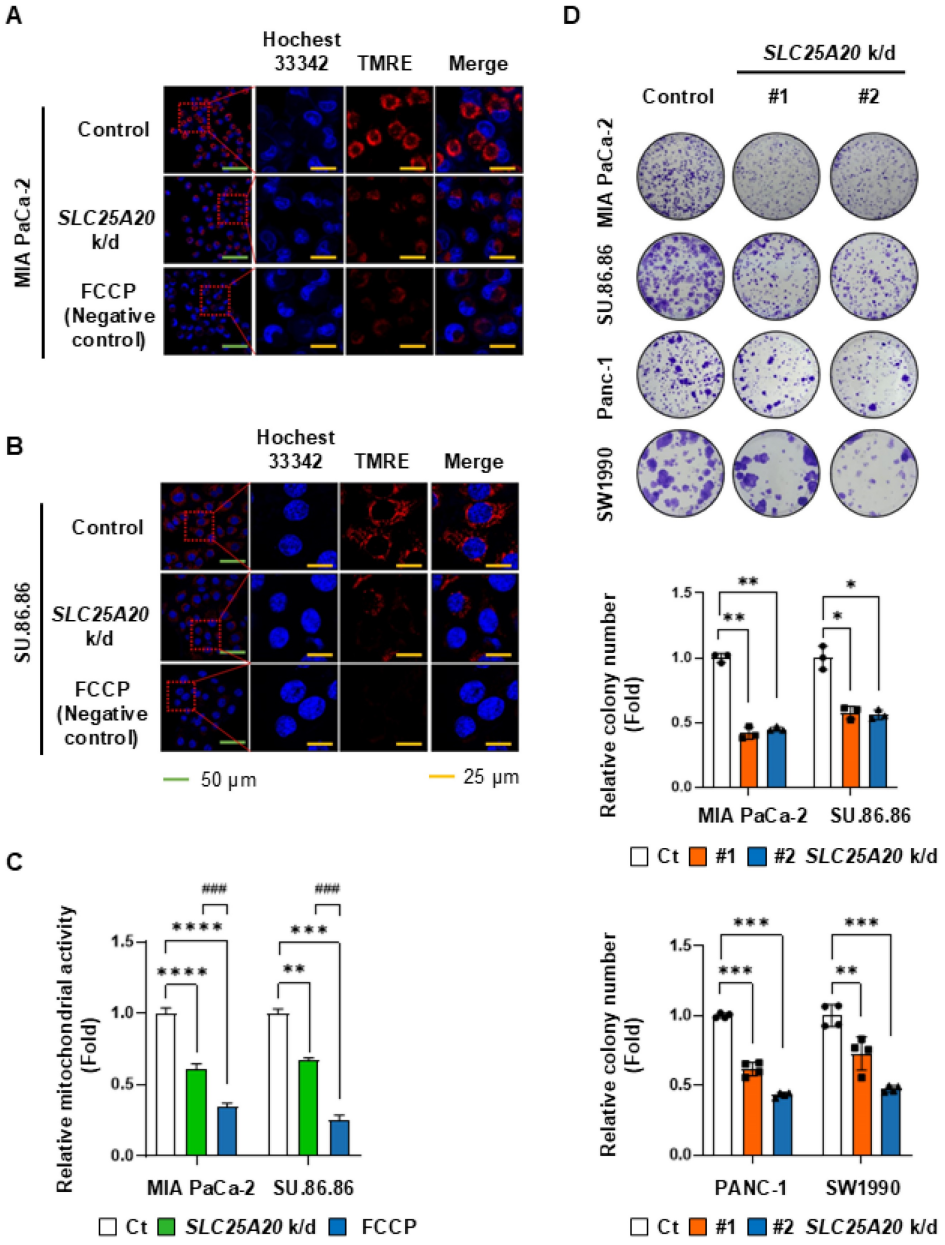
***SLC25A20* knockdown reduces mitochondrial activity and colony formation by PDAC cells. (A and B)** The mitochondrial membrane potential of MIA PaCa-2 (A) and SU.86.86 (B) without (control) and with *SLC25A20* knockdown was measured by fluorescence microscopy after TMRE staining (Red). Scale bars: green = 50 μm; yellow = 25 μm. **(C)** Mitochondrial membrane potential was assessed by measuring the intensity of TMRE fluorescence using ZEN software 3.9. The bars in the graph show TMRE intensity normalized by the number of DAPI positive per unit area. **(D)** Cancer cell growth was measured in a colony formation assay. MIA PaCa-2, SU.86.86, Panc-1 and SW1990 cells with *SLC25A20* knockdown induced by two different shRNAs (#1 and #2) were seeded in a six-well plate and cultured for 14 days. Colonies were stained with crystal violet and counted using ImageJ software. The y-axis shows the number of *SLC25A20* knockdown colonies expressed as a relative ratio, with the number of control colonies set to 1.0. Data are presented as the mean ± SD from at least three experiments. *p < 0.05, **p < 0.01, ***p < 0.001.

To evaluate whether *SLC25A20* knockdown affects mitochondrial membrane potential in pancreatic cancer cell lines, we measured the potential using TMRE (tetramethyl rhodamine ethyl ester), a cell-permeant, cationic, red-orange fluorescent dye that readily accumulates in active mitochondria. TMRE staining showed that *SLC25A20* knockdown reduced mitochondrial membrane potential in MIA PaCa-2 and SU.86.86 cells by 39% and 32%, respectively (Figure 4A-C). A colony-forming assay was performed to investigate the relationship between ATP reduction mediated by *SLC25A20* knockdown and cell growth. Colony formation by MIA PaCa-2 cells subjected to shRNA-mediated knockdown of *SLC25A20* was 55-59% lower than that of the control (Figure 4D). In SU.86.86, Panc-1, and SW1990 *SLC25A20* knockdown lines, cell growth was 44%, 48%, and 40%, respectively, lower than that in the control (Figure 4D); however, there was no change in cell proliferation in hTERT-HPNE after *SLC25A20* knockdown (Figure S6). This result is consistent with the results of a paper we recently published. We found that when pancreatic cancer cells were grown glucose free, lactate production was reduced by more than 80%, but ATP production did not change at all 14. However, when cancer cell FAO was inhibited with trimetazidine, we showed that ATP production was absolutely reduced despite the availability of all nutrients 14, 15. Therefore, the decrease in ATP with *SLC25A20* knockdown is consistent with previous results.

### PDAC cells with SLC25A20 knockdown arrest in the G1/S phase of the cell cycle following the inactivation of mTOR, with a critical impairment of the DNA repair system

**Figure 5:**
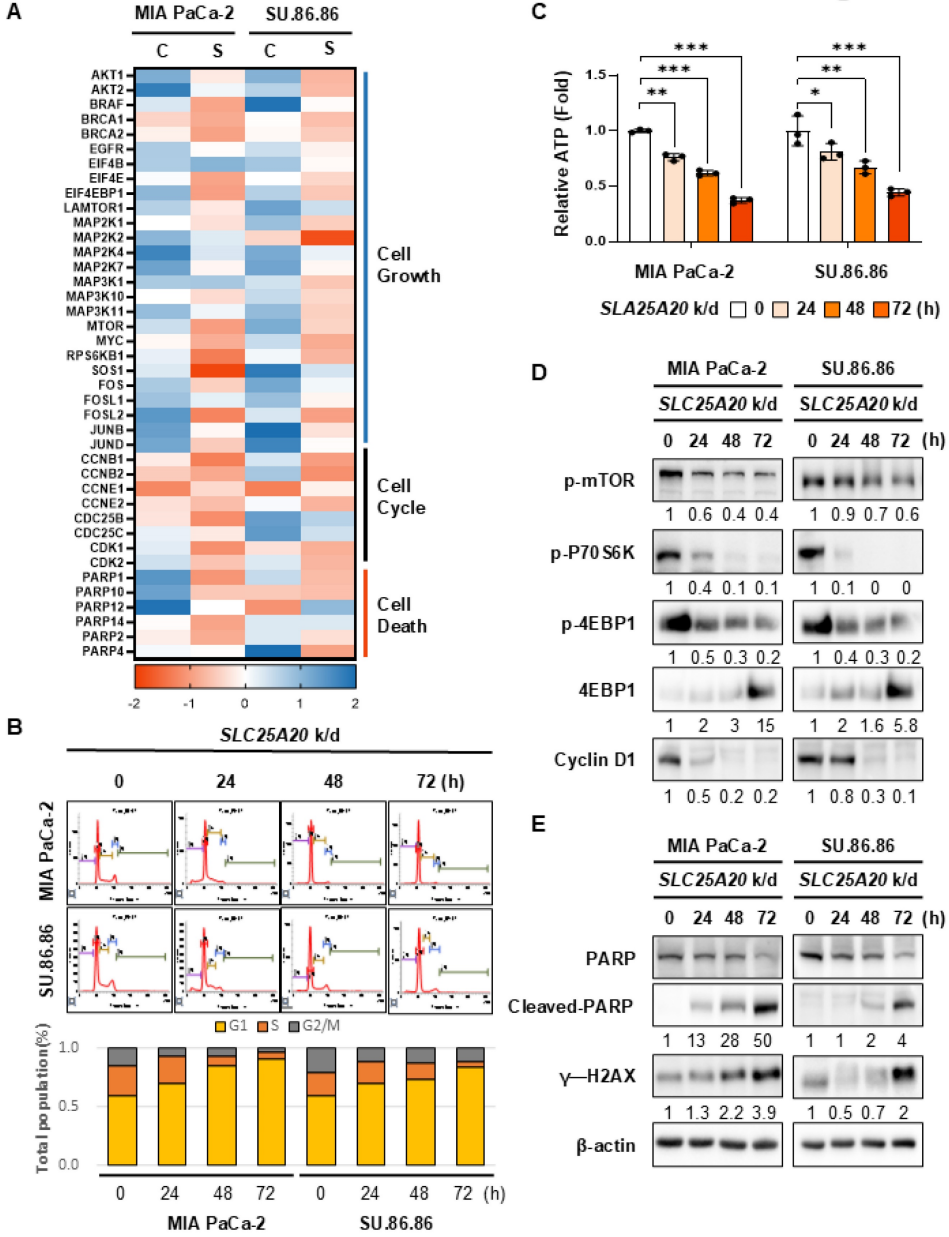
***SLC25A20* knockdown induces cell cycle arrest and cell death by turning off mTOR signaling via a reduction in ATP levels. (A)** MIA PaCa-2 and SU.86.86 were treated with 40 nM of *SLC25A20* siRNA for 48 h. LC-MS/MS was used to analyze changes in intracellular phosphor-protein levels. **(B)** Flow cytometry was used to examine the cell cycle distribution of MIA PaCa-2 and SU.86.86 cells with *SLC25A20* knockdown. Cells were synchronized to the same cycle by treatment with thymidine (2 mM) for 24 h, and then treated with siRNA at the indicated times. The cell cycle distribution was measured by staining cells with PI. **(C)** Changes in ATP production by MIA PaCa-2 and SU.86.86 cells were analyzed following *SLC25A20* knockdown. Immunoblotting was performed at different times (0-72 h post-knockdown) to analyze changes in cell growth **(D)** and cell death induction **(E)** in MIA PaCa-2 and SU.86.86 cells. The cell cycle distribution was associated with changes in cyclin D1 levels (D). DNA damage was determined by measuring changes in PARP and cleaved PARP levels. Cell death was determined by measuring changes in g-H2AX levels (E). Data are presented as the mean ± SD of at least three experiments. *p < 0.05, **p < 0.01, ***p < 0.001.

To elucidate how the reduction in intracellular ATP production following *SLC25A20* knockdown regulates cancer cell growth, we performed proteomics analysis of SU.86.86 cells using LC-MS/MS at 48 h post-knockdown (Figure 5A*,* Figure S7A). Phospho-proteomics analysis revealed that siRNA-mediated knockdown of *SLC25A20* in SU.86.86 and MIA PaCa-2 cells reduced phosphorylation of key targets involved in cell growth, including mTOR and MAPK, while phosphorylation of targets involved in cell death increased (Figure 5A). To investigate whether *SLC25A20* knockdown regulates the cell cycle, we performed cell cycle analysis in MIA PaCa-2 and SU.86.86 cells using flow cytometry (Figure 5B). *SLC25A20* knockdown using siRNA increased the G1 population from 60% to 85-90% in a time-dependent manner (from 24 to 72 h; Figure 5B), a finding that correlated inversely with ATP production following siRNA treatment (Figure 5C). ATP levels in *SLC25A20* knockdown MIA PaCa-2 and SU.86.86 cells fell by about 60% (Figure 5C). These changes corresponded with a significant decrease in cyclin D1 levels (Figure 5D, Figure S7C), which was related to the downregulation of the cell cycle in the G1/S stage 22. Blocking FAO by *SLC25A20* knockdown reduced ATP production significantly, resulting in preferential inactivation of mTOR (Figure 5D, Figure S8A). Additionally, we analyzed the global proteomic changes in pancreatic cancer cell lines treated with *SLC25A20* siRNA for 72 h. Reduced DNA repair enzymes, including excision repair cross-complementary group 1 (ERCC1), DNA mismatch repair protein Mlh1 (MLH1), DNA mismatch repair protein Msh2 (MSH2), and DNA repair protein XP-A (XPA), inactivate DNA mismatch repair, leading to single-strand breaks and increased cytotoxicity (Figure S7B). The expression of proteins in the DNA polymerase and isomerase families was also reduced. *SLC25A20* knockdown triggered cell cycle arrest and DNA damage repair, which in turn activated γ-H2AX after 72 hours (Figure 5E, Figure S8B). Regulation of protein synthesis cascades by *SLC25A20* knockdown was linked closely to the inactivation of mTOR. This implies that combining cytotoxic therapeutics with FAO inhibition may have a synergistic anti-cancer effect.

### Crossbreeding of KPC mice with Slc25a20 knock-out mice yields offspring with increased survival rates

**Figure 6:**
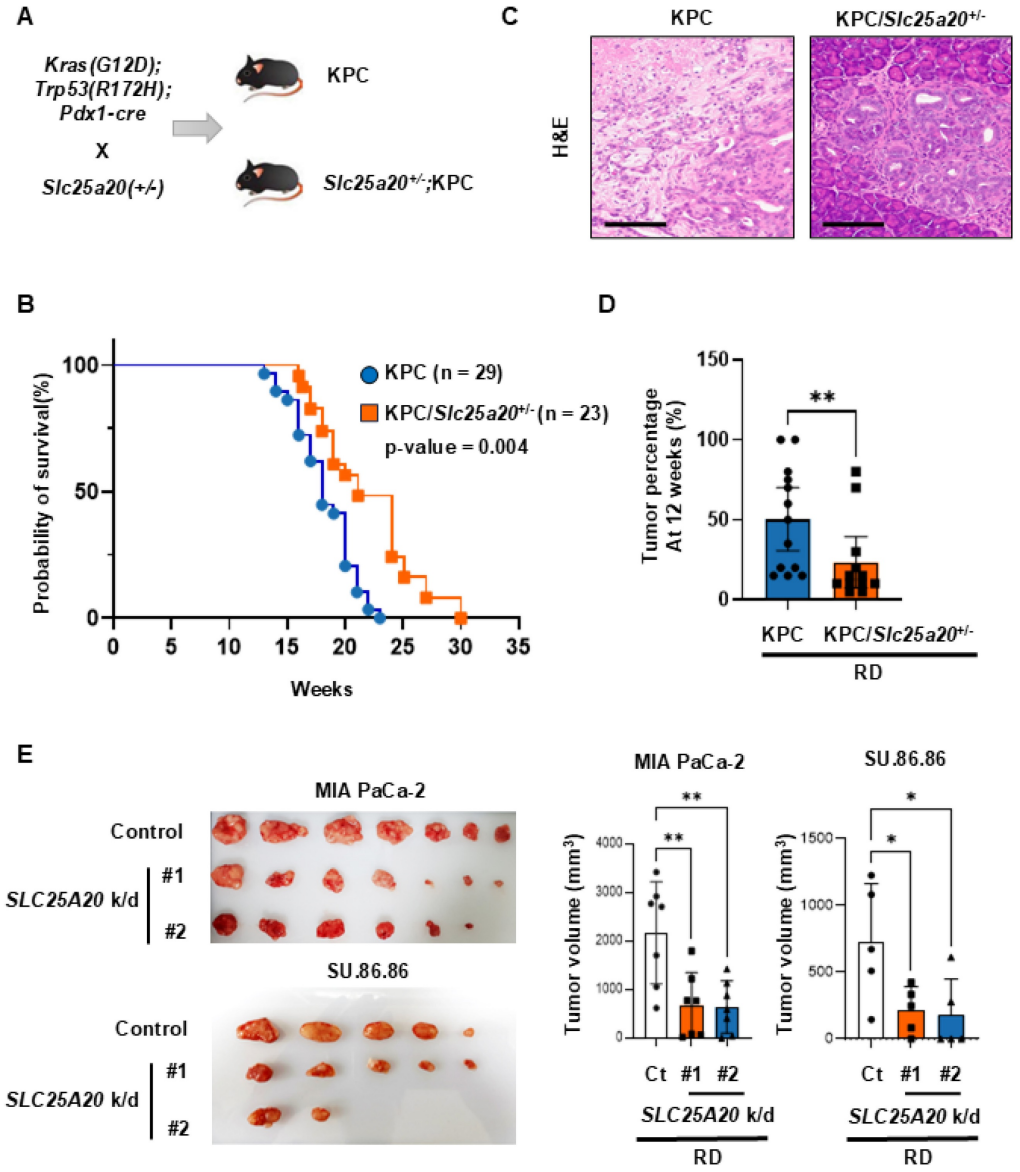
Crossing *Slc25a20* knock-out mice with KPC mice generates offspring showing slower progression of pancreatic cancer. **(A)** Crossing of KPC and *Slc25a20* knock-out mice generated KPC/*Slc25a20*^+/-^ mice harboring four mutated genes: *Kras^G12D^, Trp53^R172H^, Pdx1-cre,* and *Slc25a20*^+/-^. **(B)** Kaplan-Meier survival curves for KPC/*Slc25a20*^+/-^ mice (n = 23) and KPC mice (n = 29). The difference between the two groups was significant (p = 0.004). **(C)** In KPC/*Slc25a20*^+/-^ mice group, most mice showed sporadic changes to noninvasive, dysplastic PanIN of pancreas parenchyma (right), whereas KPC mice have invasive PDACs (left). Images of representative samples corresponding to the median values in each group (original magnification: x200, scale bar: 200μm) **(D)** Decreased PanIN and PDAC progression by the haplo-sufficiency o*f Slc25a20* in the KPC mice model. **(E)** Mouse xenograft models were tested using MIA PaCa-2 (1 x 10^7^ cells/mouse, n=7) and SU.86.86 (5 x 10^6^ cells/mouse, n =5) cells without (control) and with *SLC25A20* knockdown by two sets of shRNA #1 and #2. After 4-5 weeks of tumor growth, tumors were removed and measured in volume. The data was presented as the mean ± SD from at least three experiments. *p < 0.05, **p < 0.01, ***p < 0.001.

*Slc25a20^+/-^* mice were generated using CRISPR/Cas9 gene editing and zygote electroporation. Mutant mice harboring a 7 nt deletion in exon 2 of *Slc25a20* causes premature termination of translation, resulting in a 59-amino acid mutant protein (Figure 6A, Figure S9A & B). To investigate the role of *Slc25a20* in the development of pancreatic cancer, *Slc25a20*^+/-^ mice were mated with a *Kras*-driven pancreatic cancer model (*Kras^G12D/+;^ Trp53^R172H/+;^ Pdx1-Cre* mouse, KPC mouse) 23. When fed a RD, the median survival of the control (KPC mice) and experimental (KPC/*Slc25a20*^+/-^ mice) groups was 18 weeks and 21.1 weeks, respectively. At 20 weeks of age, approximately 60% of KPC/*Slc25a20*^+/-^ mice survived, compared with an average of 41% of KPC mice (Figure 6B). To determine if this change in survival was due to a block in fatty acid oxidation, we extracted pancreatic tissue from KPC and KPC*/Slc25a20*^+/-^ mice and measured the levels of acetyl-CoA, the end product of fatty acid oxidation, and the ATP that is produced from acetyl-CoA. The results showed that acetyl-CoA levels were reduced by 32.5% and ATP levels were reduced by 51% in pancreatic tissue from KPC/*Slc25a20*^+/-^ mice compared to KPC mice (Figure S10A-C). This is consistent with the results we demonstrated in the growth of cancer cells *in vitro.* These results indicate that blocking FAO through *Slc25a20* knockdown increases survival significantly. Pancreatic tissues were extracted from 12-week-old KPC and KPC/*Slc25a20*^+/-^ mice and subjected to H&E staining (Figure 6C, Figure S11A & B). The PanIN phenotype was predominant in KPC/*Slc25a20*^+/-^, whereas progression to PDAC was more common in KPC mice. In addition, the proportion of invasive neoplastic changes associated with PanIN and PDAC among the total pancreas parenchyma was significantly lower for KPC/*Slc25a20*^+/-^ mice than for KPC mice (Figure 6C). In the KPC group, 7/13 mice formed large tumor masses, with destruction of parenchymal lobules in the pancreas, whereas only 2/12 mice in the KPC/*Slc25a20*^+/-^ group formed large tumor masses (Figure 6D). Furthermore, immunostaining of pancreatic cancer tissues from each group revealed a 52% decrease in the expression of p-mTOR in tissues from KPC/* Slc25a20*^+/-^ mice compared to KPC mice (Figure S11C). Immunostaining could not detect the decreased expression of SLC25A20 in *Slc25a20* knockout mice (Figure S11D). However, it clearly shows that SLC25A20 knockdown in KPC significantly reduces the activity of FAO, acetyl-CoA synthesis, and ATP synthesis as well as decreased level of SLC25A20 by immunoblotting (Figure S10). In addition, a more than 2-fold reduction in p-mTOR expression was shawn in the *SLC25A20* knockdown tumor tissues (Figure S11C). Thus, the knockdown of *SLC25A20* severely reduces the activity of mTOR due to a decrease in FAO-dependent ATP, and these changes are a major contributor to invasive tumor changes and reduced progression to PDAC. We also knocked down *SLC25A20* in SU.86.86 and MIA PaCa-2 cancer cells using shRNA and then implanted the cells into mice fed an RD (Figure 6E). Two knockdown cell lines from each group were selected and inoculated into mice, and tumor size was measured over time. MIA PaCa-2 with *SLC25A20* knockdown formed tumors that were 70%-80% smaller than those in the control group, and SU.86.86 cells with *SLC25A20* knockdown formed tumors that were 70%-75% smaller (Figure 6E).

### A preclinical mouse model reveals that SLC25A20 knockdown suppresses tumor growth promoted by an HFD

**Figure 7:**
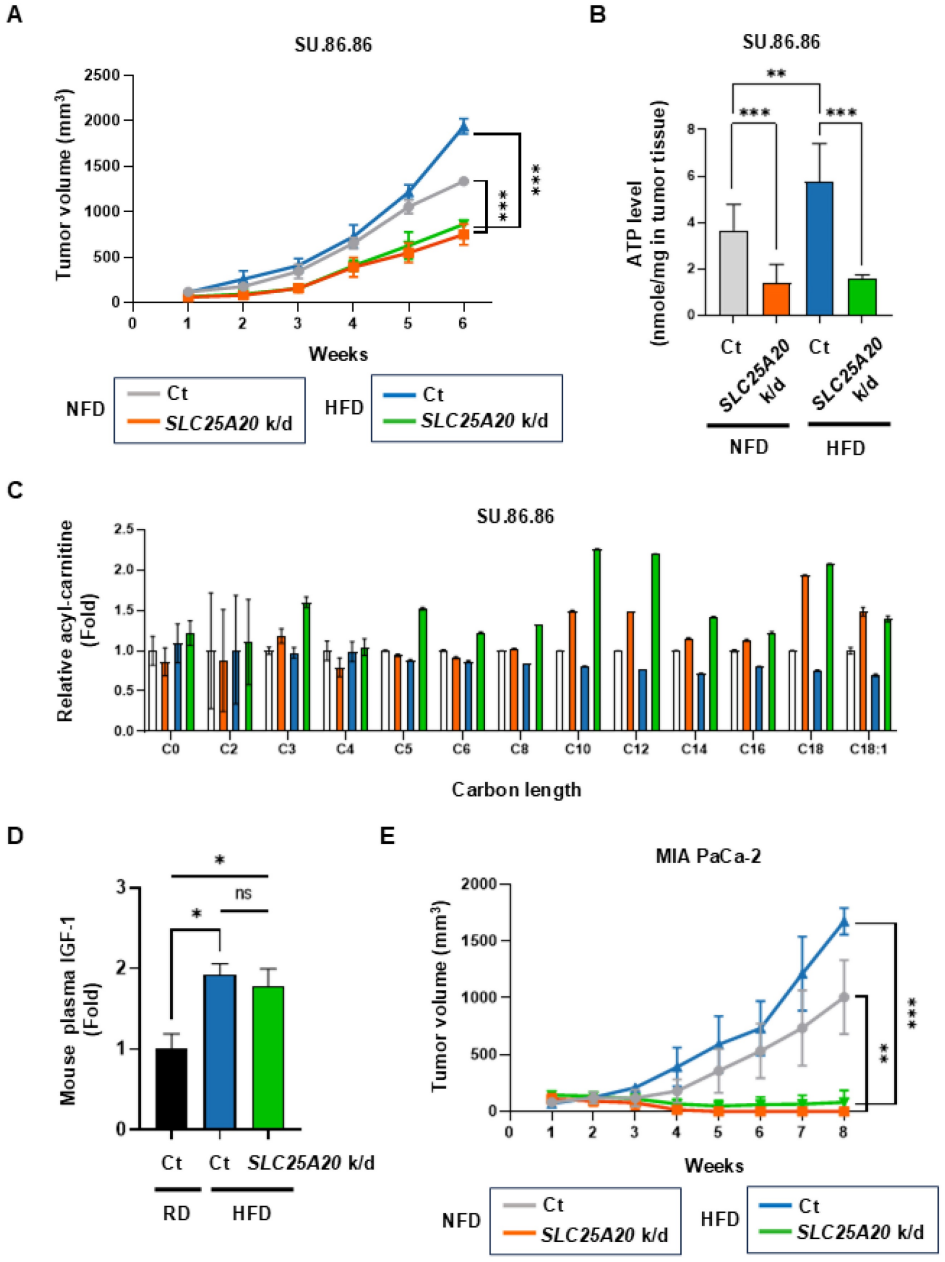
*SLC25A20* knockdown slows the growth of HFD-induced pancreatic cancer. **(A)** The effect of *SLC25A20* knockdown in the SU.86.86 xenograft model (5 × 10^6^ cells/mouse; n=10) mice fed a caloric-balanced HFD (60% fat) or NFD (0% fat). Growth of *SLC25A20* knockdown (orange) cells was compared with that of control (gray) cells under NFD conditions. Growth of *SLC25A20* knockdown (green) cells was compared with the control (blue) under HFD conditions. **(B)** Intra-tumor ATP levels in SU.86.86 *SLC25A20* knockdown tumor and control tissues. **(C)** Blood acyl-carnitine concentrations were analyzed in the control and *SLC25A20* knockdown SU.86.86 groups. **(D)** The amount of IGF-1 in plasma was measured to investigate the pathways driving tumor growth. **(E)** The effect of *SLC25A20* knockdown in the MIA PaCa-2 xenograft model (5 × 10^6^ cells/mouse; n=6) was also tested in mice fed a calorie-balanced HFD or NFD. Growth of *SLC25A20* knockdown (orange) cells compared with the control (gray) under NFD conditions, and growth of *SLC25A20* knockdown (green) cells compared with the control (blue) under HFD conditions. Statistical analysis was performed using unpaired two-tailed t-tests to compare differences between groups. Data are presented as the mean ± SD from at least three experiments. *p < 0.05, **p < 0.01, ***p < 0.001.

Based on the above results, it seems that the increased supply of ATP via FAO in tumor cells is directly responsible for driving the growth of cancer cells. Therefore, we knocked down *SLC25A20* in cancer cells to inhibit FAO and tested whether tumor growth driven by an HFD is inhibited. SU.86.86 and MIA PaCa-2 cells were subjected to *SLC25A20* knockdown using shRNA and then implanted into mice fed a caloric-balanced HFD (60% fat) or NFD (0% fat) (Figure 7A & E). Tumor size was measured over time. Tumors in the *SLC25A20* knockdown fed were 45% smaller than those in the controls. Also, the tumors in the control HFD group were 30% larger than those in the control NFD group (Figure 7A). In the HFD group, *SLC25A20* knockdown reduced tumor size by about 65% compared with the HFD control group (Figure 7A). Tumor growth in the NFD and HFD *SLC25A20* knockdown groups was quite similar (Figure 7A), suggesting that HFD-induced tumor promotion is completely dependent on FAO. Next, we collected tumor and blood samples from mice implanted with SU.86.86 cells (Figure 7B-D). The tumors from mice yielded approximately ~100 μg of protein. Therefore, we had to choose one of the two experiments. One is extracting ATP from the tissue and quantifying it, while the other one is metabolite analysis. However, given the importance of the experiment, we decided that ATP measurement was essential and proceeded with that (Figure 7B). ATP levels in the HFD control group increased by 58% compared with the NFD control group, whereas those in the NFD *SLC25A/20* knockdown group decreased by 63% compared with the NFD control group, and those in the HFD SLC25A/20 knockdown group decreased by 74% compared with the HFD control group (Figure 7B). Next, we examined acyl-carnitine levels by conducting an M/S analysis of the tumors. The *SLC25A20* knockdown group fed an NFD showed an increase in acyl-carnitines C10-C18 levels, while the *SLC25A20* knockdown group fed an HFD showed an increase of acyl-carnitines C3-C18 (Figure 7C). The levels of C10 and C12 increased by > 2-fold in the HFD *SLC25A20* knockdown group compared with the HFD control (Figure 7C). This implies that cancer cells depend on medium chain (MC) fatty acids for FAO to generate ATP and that MC fatty acids translocate to the mitochondria via *SLC25A20*. HFD-mediated hyperinsulinemia increases IGF-1 levels 9. In this context, we found that an HFD increased IGF-1 levels by 1.9-fold when compared with an RD (Figure 7D). The HFD *SLC25A20* knockdown group also showed a >2-fold increase in IGF-1 levels when compared with the control group. This implies that HFD-driven tumor growth depends not only on IGF-1 signaling but on FAO pathways. We also examined the effect of *SLC25A20* knockdown using MIA PaCa-2 cells transplanted into mice fed an HFD or NFD (Figure 7E). Tumors in the *SLC25A20* knockdown group fed an NFD were 99% smaller than those in the control. In the control group they fed an HFD, tumors grew 60% larger than those in the control group which fed an NFD (Figure 7E). Finally, in the HFD group, *SLC25A20* knockdown reduced tumor size by 95% compared with that in the HFD control group (Figure 7E). Tumor growth in the *SLC25A20* knockdown groups fed an NFD or HFD was almost the same (Figure 7E).

## Discussion

**Figure 8:**
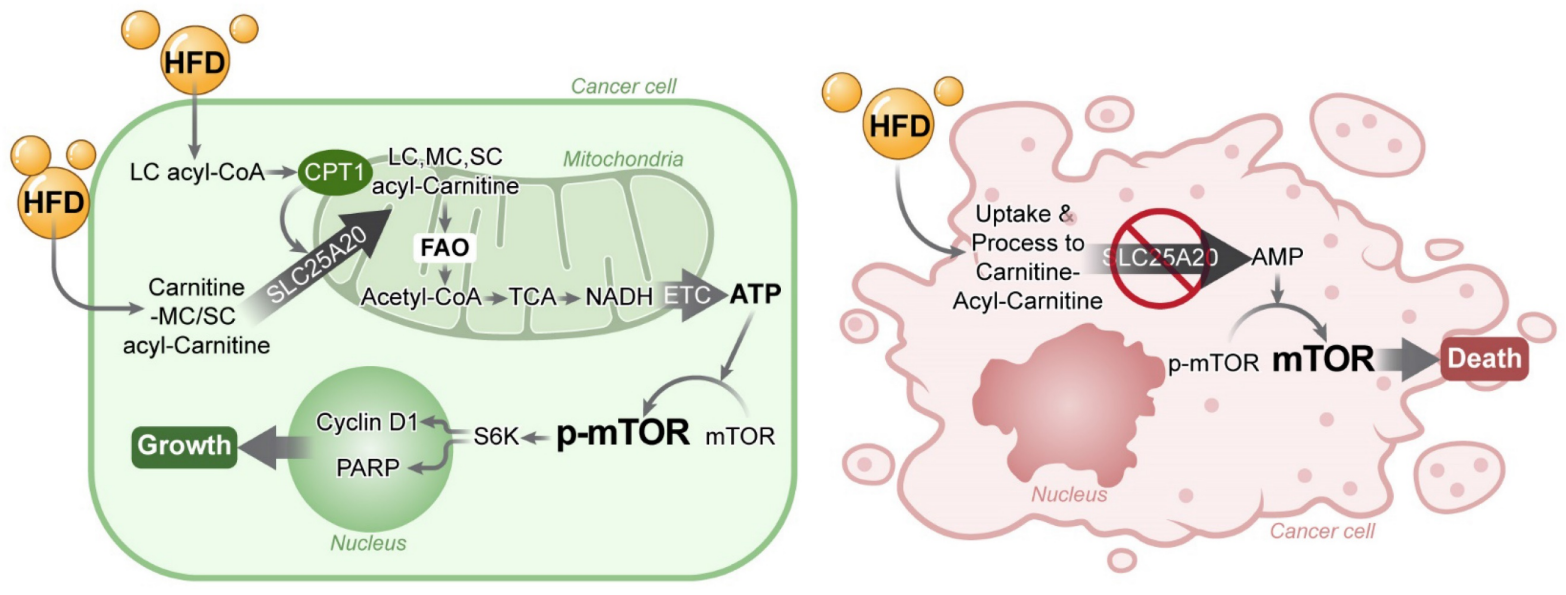
Blocking FAO by *SLC25A20* knockdown reversed HFD-induced tumor promotion. **(A)** Cancer cells absorb fatty acids from HFDs and convert them into LC, MC, and SC acyl-carnitine, which they use to produce acetyl-CoA via FAO in the mitochondria. The acetyl-CoA is converted to NADH in the TCA cycle, which is then used to produce ATP via the electron transport chain (ETC). Previously this FAO dependent ATP production in cancer was proposed as “Kim effect” to avoid confusion with the reprogramming theories 17. Increased ATP levels activate p-mTOR, which in turn increases the expression of cyclin D1, essential for cell growth, and promotes the expression of PARP, which plays an important role in detecting and repairing DNA damage within cells. These survival programmes promote the growth of cancer cells. **(B)** FAO inhibition by *SLC25A20* knockdown caused a decrease in ATP level, which induced mTOR inactivation, and consequently resulted in cell cycle arrest and cell death activation.

Cancer cells depend on FAO for ATP production; therefore, blocking FAO results in cell growth arrest. Here, we found that mTOR is a very sensitive switch that is turned on or off according to the level of ATP generated by FAO. HFD increases FAO, which in turn increases ATP and, consequently, mTOR activation, this drives cancer growth. Here, we show that knocking down *SLC25A20* in PDAC cells prevents HFD-dependent tumor growth in a xenograft model (Figure 8).

A previous report shows that the mTOR pathway is regulated by the intracellular level of ATP, a phenomenon that is independent of the abundance of amino acids; this suggests mTOR itself acts as an ATP sensor 24. The *K*m value (Michaelis Constant) of mTOR for ATP is about 1.0 mM, tens to hundreds of times higher than that of other kinases 24-26. This implies that it is activated at high ATP concentrations and inactivated when the intracellular level of ATP drops even slightly below 1.0 mM. Indeed, mTOR is regulated directly by the intracellular ATP concentration, although mTOR is also known to be regulated by amino acids 24. The intra-tumor level of ATP measured in the xenograft model showed an approximate 50% decrease in ATP production by PDAC cancer cells in which *SLC25A20* was knocked down. mTOR acts as the master regulator of cell metabolism by controlling responses to various metabolic signals.

We observed that knockdown of the FAO gene, or inhibition by high glucose cultures, reduced ATP production by cancer cells significantly, which is consistent with previous observations of increased CPT1A expression in cancers 27. Furthermore, we found here that HFD-induced cancer cell outgrowth or tumor promotion was abrogated completely by the knockdown of the FAO gene in PDAC cells. Fatty acids are oxidized via FAO to yield TCA intermediates, which then generate intermediates of various amino acids; further oxidation generates ATP through an electron transfer chain using NADH and FADH 28. This suggests that the TCA-ETC-OxPhos system (tricarboxylic acid cycle-electron transfer complex-oxidative phosphorylation) functions properly in cancer cells 28. We reported previously that most cancer cells depend on FAO to generate ATP 16, 17. Recent clinical trials focused on strategies that inhibit fatty acid transporters. One target of fatty acid transport is CPT1A, which transports fatty acids from the cytoplasm to the mitochondria. Although CPT1A is expressed in normal cells, cancer cells show unusually high expression 27, 29. The reason why research on SLC25A20 and the development of inhibitors have been behind the priority is that FAO of cancer cells was considered to be the same as that of normal cells, leading to CPT1A being targeted as the primary target. To inhibit FAO in normal cells, targeting CPT1A in mitochondria is sufficient because normal cells primarily utilize long chain (LC) acyl-carnitine for FAO. Therefore, CPT1A inhibitors such as etomoxir 30, Perhexiline 31, Teglicar (ST1326) 32 have been extensively studied and are currently undergoing clinical trials, whereas SLC25A20 inhibitors have no known clinical outcomes. However, as demonstrated in this study, cancer cells utilize SC/MC acyl-carnitines extensively, suggesting that these acyl-carnitines bypass CPT1A and directly enter mitochondria via SLC25A20 20. Thus, in cancer cells, SLC25A20 has shown potential to inhibit FAO as an independent target of CPT1A, emerging as a promising strategy to inhibit FAO.

In addition to the mechanism by which HFD directly promotes ATP production in tumors, various studies are underway to understand the molecular mechanisms by which HFD promotes tumor growth. Epigenetic and transcriptomic changes associated with tumor growth promotion, including metabolic and immune-related pathways, were observed in the intestines of genetically tumor-prone mice induced by a 3-day HFD, but these changes were found not to affect major cancer signal transduction pathways 33, 34. However, the dependence of ATP synthesis in cancer cells on FAO may not be due to complex genetic or non-genetic regulation, but rather due to a very simple reason. Cancer cells may have become highly dependent on FAO for ATP production because the Warburg effect blocked common source of energy, glucose, leaving them with no choice but to rely on FAO to compensate, much like a hibernating squirrel 35.

When FAO is inhibited in cancer cells, glucose continues to be converted into lactic acid, and if glutamine is used as an energy source instead of glucose and fatty acids, ATP synthesis should remain unchanged. However, when fatty acid oxidation is inhibited in cancer cells, ATP synthesis is severely reduced. Glutamine conversion into the TCA cycle intermediate α-ketoglutarate via glutamate,2 which is catalyzed by GLS1 and glutamate dehydrogenase, is also essential for Kras-induced anchorage-independent growth 36. This suggests that the production of α-ketoglutarate (α-KG) catalyzed by GLS1 and glutamate dehydrogenase is further catabolized to citrate, which turns into acetyl-CoA for fatty acid synthesis in glioblastoma cells 37. Based on previous reports, glucose and glutamine are considered to support cancer cell anabolism instead of energy metabolism 37, 38. Using [^13^C]glucose labeling, studies of melanoma and glioblastoma showed 75~93% of glucose was catalyzed to lactate 37, 38. A study using [^13^C]glucose labeling showed that small amount of pyruvate from glycolysis produced oxaloacetate by pyruvate carboxylase 36. Glutamine is a known alternate carbon source for cancer cells. Therefore, metabolite flux study using [^13^C]glutamine and [^13^C]glucose showed that glucose was the primary carbon source for glycolytic intermediates as well as source for serine and glycine while glutamine did not contribute to glycolysis at all 38. However, glutamine was the primary carbon source for TCA cycle intermediates through α-KG as well as source for proline and aspartic acid in melanoma and GBM 37, 38. Citrate is exported from mitochondria to the cytosol where it is converted to acetyl-CoA which can be a source for fatty acid synthesis. Therefore, ^13^C-labeling study showed the majority of fatty acids showed ^13^C-labeling from glucose and glutamine 37, 38. This suggests that glucose and glutamine are not burned in the mitochondria to produce energy and become CO_2_. They are used to provide building blocks rather than energy sources in cancer cells.

In summary, when FAO is blocked, ATP production is significantly inhibited in PDAC cells. Targeting FAO will be a novel therapeutic approach and promising particularly due to the metabolic dependence of PDAC cells, but for a rapid therapeutic response in pancreatic cancer, FAO inhibitors are expected to be most effective when used in combination with other anticancer drugs. Continued research is necessary to better understand how to exploit FAO inhibition for therapeutic gain. This is because most anti-cancer therapies target anabolic metabolic pathways, suggesting that a catabolic inhibitor targeting FAO would have a significant synergistic effect when used alongside standard anti-cancer drugs. Another expectation is that inhibitors targeting FAO will increase responsiveness to standard anti-cancer drugs, as resistance to anti-cancer treatment is associated with increased levels of FAO 39.

## Materials and Methods

### Cell culture

The hTERT-immortalized pancreas epithelial cell line hTERT-HPNE (CRL-4023) was purchased from ATCC (Manassas, VA, USA) and grown in 75% glucose-free DMEM (D-5030, Sigma-Aldrich, St. Louis, MO, USA) supplemented with 2 mM L-glutamine and 1.5 g/L sodium bicarbonate, plus 25% Medium M3 Base (Incell Corp. Texas, USA) containing 5% fetal bovine serum, 5.5 mM D-glucose (G8270, Sigma-Aldrich), and human recombinant EGF (E9644, Sigma-Aldrich). MIA PaCa-2, PANC-1, and SW1990 cells were grown in DMEM high glucose medium (SH30243.01, Cytiva, Logan, UT, USA) containing 10% fetal bovine serum and penicillin/streptomycin. SU.86.86 cells were grown in RPMI 1640 medium containing 10% fetal bovine serum and penicillin/streptomycin. All cells were maintained at 37°C and 5% CO_2_ humidity.

### Animal studies

Balb/c-nu/nu mice (Orient, Seoul, Korea, aged 6-8 weeks) were used for the mouse xenograft study using human PDAC cells. This study was reviewed and approved by the Institutional Animal Care and Use Committee of the National Cancer Center Research Institute (protocols: NCC-21-661, NCC-24-1055). MIA PaCa-2 cells (1 × 10⁷) and SU.86.86 cells (5 × 10⁶) were mixed with Matrigel (BD Biosciences, Franklin Lakes, NJ, USA) at a 1:1 ratio and subcutaneously injected into mice. To analyse the anti-tumour effects targeting FAO, mice injected with pLKO.1-puro_scramble shRNA or pLKO.1-puro_SLC25A20 shRNA (shRNA#1 or #2) were divided into two groups and fed either NFD (D04112303, Research Diets, New Brunswick, NJ, USA) or a high-fat diet (HFD, D12492, Research Diets, New Brunswick, NJ, USA). The anti-cancer effects of suppressing the *SLC25A20* gene in combination with the low-fat or high-fat dietary regimens were then evaluated. The size of the primary tumor was measured weekly using calipers. Tumor volume was calculated using the following formula: V = (A × B^2^)/2, where V is the volume (mm^3^), A is the long diameter, and B is the short diameter.

*Slc25a20* knockout mice were generated using the CRISPR/Cas9 genome editing technology. The target sequences for the single guide RNA (sgRNA) were selected using the CRISPR design tool (crispor.tefor.net). A mixture of Cas9 protein (100 ng/mL) and sgRNA (50 ng/mL) was transferred to mouse embryos through zygote electroporation 40. The target sequences for CRISPR/Cas9 gene editing were 5'-GGC TGT CCA GAC AAA CTT GG-3', 5'-AAG GTC CCA GAG TAC ATA GG-3'. Indel mutations in F1 mice were identified after TA cloning and sequencing. The Cas9 protein (EnGen Cas9 NLS) was purchased from NEB, and sgRNAs were generated using a T7 *in vitro* transcription kit (NEB).

The spontaneous pancreatic cancer animal model mouse *Kras^G12D^*; *Trp53^R172H^*; and *Pdx1-Cre* (KPC) was used to investigate the impact of *Slc25a20* on the development and progression of pancreatic cancer. The KPC mouse model has been reported previously 41, but a brief explanation will be provided here. *Kras^G12D^* mice (B6.129-Kras^tm4Tyj^/Nci) and *Trp53^R172H^* mice (124S4-Trp53^tm2Tyj^/Nci) were obtained from the NCI Mouse Repository. *Pdx1-Cre* mice (B6.FVB-Tg(Pdx1-Cre)6Tuv/J, #014647) were purchased from the Jackson Laboratory. *Kras^G12D^; Pdx1-Cre* (KC) mice were generated by crossing *KrasG12D* mice with *Pdx1-Cre* mice. *Kras^G12D^; Trp53^R172H^; Pdx1-Cre* (KPC) mice were obtained by crossing KC mice with *Trp53^R172H^* mice. *Slc25a20* hetero knockout KPC mice were generated by crossing KC mice with *Slc25a20* heterozygous mice to obtain *Kras^G12D^; Pdx1-Cre; Slc25a20^+/-^* mice. The *Trp53^R172H^* mice were then crossed with* Slc25a20^+/-^* mice to obtain *Trp53^R172H^; Slc25a20^+/-^* mice which were then crossed with KC; *Slc25a20^+/-^* mice to generate *KPC; Slc25a20^+/-^* mice*.* The study was reviewed and approved by the Institutional Animal Care and Use Committee (IACUC) of the National Cancer Center Research Institute (protocols: NCC-21-574B).

To evaluate the impact of fatty acid intake on the development and progression of pancreatic cancer, KPC mice were divided into two groups: a control group fed a regular diet (1314 IRR, Altromin GmbH, Lage, Germany) and a high-fat diet group (D12492, Research Diets, New Brunswick, NJ, USA). Survival rates were compared. The study was reviewed and approved by the Institutional Animal Care and Use Committee (IACUC) of the National Cancer Center Research Institute (protocols: NCC-24-1051), which is an Association for Assessment and Accreditation of Laboratory Animal Care International (AAALAC International) accredited facility that abides by the Institute of Laboratory Animal Resources guide.

### Human subjects

A pancreatic cancer tissue microarray (TMA, PA2072a) was purchased from Tissue Array (Derwood, MD, US). Tissue microarray (TMA) is exempt from review by the National Cancer Center Institutional Review Board (IRB) under the following conditions: TMAs are used for research purposes, and samples are provided in an anonymous or unidentifiable form. The TMAs we analyzed were provided anonymously by a US supplier and are therefore exempt from IRB review. Analysis of data from patients with pancreatic cancer: The expression level of CPT1A in pancreatic adenocarcinoma (PAAD) and matched normal control samples was analyzed using the GEPIA webserver (http://gepia.cancer-pku.cn/). Tumor data were obtained from the TCGA (n = 179), and normal data were obtained from both the TCGA and GTEx (n = 171). The relationship between CPT1A expression levels and survival of PAAD patients was analyzed using the Kaplan-Meier Plotter (https://kmplot.com/analysis). This online tool integrates gene expression data and survival information from publicly available databases (GEO, EGA, and TCGA) to assess the prognostic value of gene expression on patient survival. Kaplan-Meier survival curves were generated to compare the overall survival (OS) and relapse-free survival (RFS) rates between the high and low-expression groups. The hazard ratio with 95% confidence intervals, and the log-rank p-value, were calculated to determine the statistical significance of the differences.

### Immunohistochemical staining

Immunohistochemistry (IHC) was performed on formalin-fixed paraffin-embedded (FFPE) pancreas tissues from mice and TMA sections. The primary antibodies used in this experiment, their sources, and dilution ratios were as follows: CPT1A (Cat No. ab234111, 1:500), CK19 (Cat No. ab52625, 600:1) from Abcam (Cambridge, UK) and Phospho-mTOR (Ser2448) (D9C2) (Cat No. 5536S, 1:25) from Cell Signaling Technology (Danvers, MA, US).

### Image acquisition and analysis

The stained human pancreatic cancer TMA and tissue sections from the mouse model were scanned in high-resolution mode using the Vectra Polaris multispectral imaging system (Akoya Biosciences, Marlborough, MA, US) and Motic Easy Scan digital slide scanner (Motic, Kowloon Bay, Hong Kong). Protein expression was analyzed based on the pathological evaluation of tissue morphology and staining patterns in pancreatic cancer tissues. The inForm Image Analysis Software (Akoya Biosciences, Marlborough, MA, US) was employed to assess the IHC results quantitatively. H-scores were calculated based on the percentage of positively stained cells and staining intensity. The significance of CPT1A expression across different groups was analyzed using one-way ANOVA in GraphPad Prism version 10.3.1.

### *In vitro* colony formation assay

To evaluate the effects of increased fatty acid supply or inhibition of fatty acid oxidation on pancreatic cancer cell growth, a Colony Formation Assay was performed by the established protocol 42. Each experiment was performed in triplicate, and the growth of pancreatic cancer cell lines under different experimental conditions was analyzed using one-way ANOVA in GraphPad Prism version 10.3.1.

### Oxygen consumption rate analysis

To evaluate changes in cellular oxygen consumption rate and ATP production following increased intracellular fatty acid uptake and inhibition of fatty acid oxidation, an Oxygen Consumption Rate (OCR) analysis was performed by the established protocol 41. Seahorse XFe96/XF Pro Cell Culture Microplates (Cat No. 103794-100), Seahorse XF Cell Mito Stress Test Kit (Cat No. 103015-100), and Seahorse XF Calibrant Solution (Cat No. 100840-100) were purchased from Agilent (Santa Clara, CA, US). To normalize cell number per well, the Sulforhodamine B (SRB) assay was employed 43, 44. The experiments were conducted in triplicate, and statistical significance between the control and experimental groups was analyzed using one-way ANOVA with GraphPad Prism version 10.3.1.

### Mitochondrial activity staining (TMRE)

Mitochondrial membrane potential was analyzed by measuring tetramethylrhodamine-ethyl ester (TMRE, 87917, Sigma-Aldrich, St Louis, US) staining. The assay method was followed as previously published 41. Live cell imaging was performed using the LSM780 Laser Scanning Microscope and Axio Observer Z1 (Carl Zeiss, Oberkochen, Germany). The relative intensity of TMRE was normalized by the arithmetic mean intensity (from ZEN software 3.4). The experiments were conducted in triplicate, and statistical significance between the control and experimental groups was analyzed using one-way ANOVA in GraphPad Prism version 10.3.1.

### Glutamine treatment effect on ATP level

Cells were transfected with scramble siRNA or *SLC25A20* siRNA (final concentration 40 nM) in 60 mm dishes for 48 hours. The transfected cells were seeded into XFe 96 well microplates at a density of 20,000 cells per well, and measured OCR followed by dose-dependent treatment with L-Glutamine (Cat. No. G8540, Sigma-Aldrich, St. Louis, USA) for 24 hours.

### Quantitation of metabolites of acyl-carnitine using liquid chromatography-tandem mass spectrometry (LC-MS/MS)

Metabolites were analyzed with LC-MS/MS equipped with a 1290 HPLC (Agilent, Santa Clara, CA, US), Qtrap 5500 (AB Sciex, Vaughan, CA, US), and an LC column as we have published before 16.

### Cell cycle analysis

To evaluate the effect of SLC25A20 inhibition on the cell cycle, flow cytometry (FACS) analysis was performed. The assay method followed was as previously published 45. The experiments were conducted in triplicate, and statistical significance between the control and experimental groups was analyzed using one-way ANOVA in GraphPad Prism version 10.3.1.

### Establishment of pancreatic cancer cell lines with stable knockdown of *SLC25A20* using lentivirus

Lentiviral transduction was employed to establish pancreatic cancer cell lines with stable knockdown of the *SLC25A20* gene by the established protocol 16. Lentiviral *SLC25A20* shRNA plasmids (TRCN0000291448, TRCN0000307710) were purchased from Sigma-Aldrich (St. Louis, Missouri, US). To investigate intracellular signaling and metabolite profile changes induced by *SLC25A20* knockdown using siRNA, *SLC25A20* siRNA (Cat No. s535090) was purchased from Thermo Fisher Scientific (Waltham, MA, US). Various cancer cell lines, including pancreatic cancer (MIA PaCa-2, SU.86.86, SW1990, PANC-1), glioblastoma (T98G), breast cancer (MDA-MB-231), prostate cancer (PC-3), non-small cell lung cancer (A549), and colorectal cancer (HCT 116), were purchased from the ATCC (Manassas, VA, US). Cells were transfected with either negative control siRNA or *SLC25A20* siRNA using Lipofectamine 3000 reagent (Cat No. L3000075; Thermo Fisher Scientific, Waltham, MA, US) according to the manufacturer's instructions.

### Immunoblotting

To investigate changes in intracellular signaling pathways following the modulation of fatty acid oxidation targets, immunoblotting was performed by the established protocol 16. The primary antibodies used in this experiment, their sources, and dilution ratios were as follows: SLC25A20 (Cat No. ab244436, 1:500), CK19 (Cat No. ab52625, 600:1) and CPT1A (Cat No. ab128568, 1:1000) from Abcam (Cambridge, UK); β-actin (Cat No. sc-47778, 1:1000) and Topo I (Cat No. sc-5342, 1:1000) from Santa Cruz Biotechnology (Dallas, TX, US); and various antibodies from Cell Signaling Technology (Danvers, MA, US), including mTOR (Cat No. 2972S, 1:1000), Phospho-mTOR (Ser2448) (Cat No. 2971S, 1:1000), Cyclin D1 (Cat No. 2978S, 1:1000), PARP (Cat No. 9572S, 1:1000), Cleaved PARP (Cat No. 9541S, 1:1000), eIF4E (Cat No. 9742S, 1:1000), p70S6 Kinase (Cat No. 9202S, 1:1000), Phospho-p70 S6 Kinase (Thr389) (Cat No. 9234S, 1:1000), 4E-BP1 (Cat No. 9452S, 1:1000), Phospho-4E-BP1 (Thr37/46) (Cat No. 9459S, 1:1000) and gamma-H2AX (Cat No. A300-081A, 1:1000) from FORTIS Life Sciences (Waltham, MA, US).

### Measurement of ATP levels

ATP levels in tumor tissue were measured using an ATP Colorimetric/Fluorometric Assay Kit (ab83355, Abcam, Cambridge, UK). The assay method was followed as previously published 18.

### IGF-1 assay

Mouse IGF-1 levels were measured using a Mouse IGF-1 ELISA assay kit (ab100695; Abcam, Cambridge, UK) by the manufacturer's procedures. Blood samples were collected from xenograft mice via intracardiac bleeding. The blood was centrifuged at 2000 rpm for 20 minutes at 4°C to isolate the serum, collected, and stored at -80°C until further use. Serum samples were diluted 1:100 before use in the assay. A paired t-test was conducted for statistical analysis.

### Acetyl-CoA assay & lactate assay

The levels of acetyl-CoA were measured using the acetyl-CoA assay kit (Abcam, Cat. No. ab87546) and lactate levels were measured using the L-Lactate assay kit (ab65330, Abcam) according to the manufacturer's instructions.

### Statistical analysis

All statistical analyses were performed using the GraphPad Prism 10 software (GraphPad Software Inc., San Diego, CA, USA). Survival rates were calculated using the Kaplan-Meier method and compared using the log-rank test. Differences were analyzed by one-way analysis of variance (ANOVA). P values *< 0.05, **< 0.01, ***< 0.001, and ****< 0.0001 were considered significant.

## Supplementary Material

Supplementary figures.

## Abbreviations

PDAC: pancreatic ductal adenocarcinoma
NADH: nicotinamide adenine dinucleotide
ATP: adenosine triphosphate
also known as the Krebs cycle): TCA cycle, tricarboxylic acid cycle
FAO: fatty acid oxidation
KPC mouse: *Kras^G12D/+;^ Trp53^R172H/+;^ Pdx1-Cre* mouse
CACT): SLC25A20, carnitine-acylcarnitine carrier (CAC
