# Supplementary material for: Loss of SLC25A20 in Pancreatic Adenocarcinoma Reversed the Tumor-Promoting Effects of a High-Fat Diet
Source: Theranostics. 2025 May 25;15(13):6516–33. doi: 10.7150/thno.114912 (PMC12160018; doi:10.7150/thno.114912)
Supplement: Supplementary file 1 — Supplementary figures. [file thnov15p6516s1.pdf]

Supplementary Figure S1

A

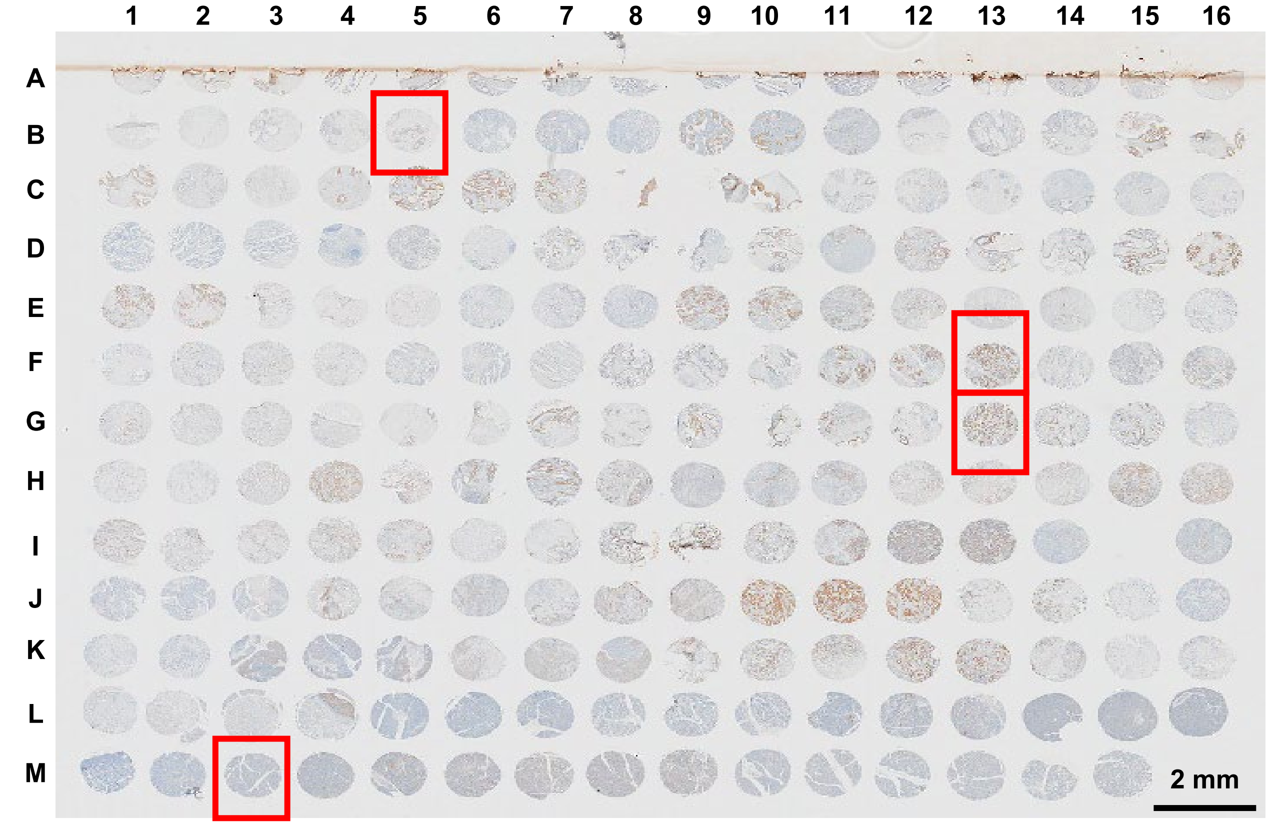

B

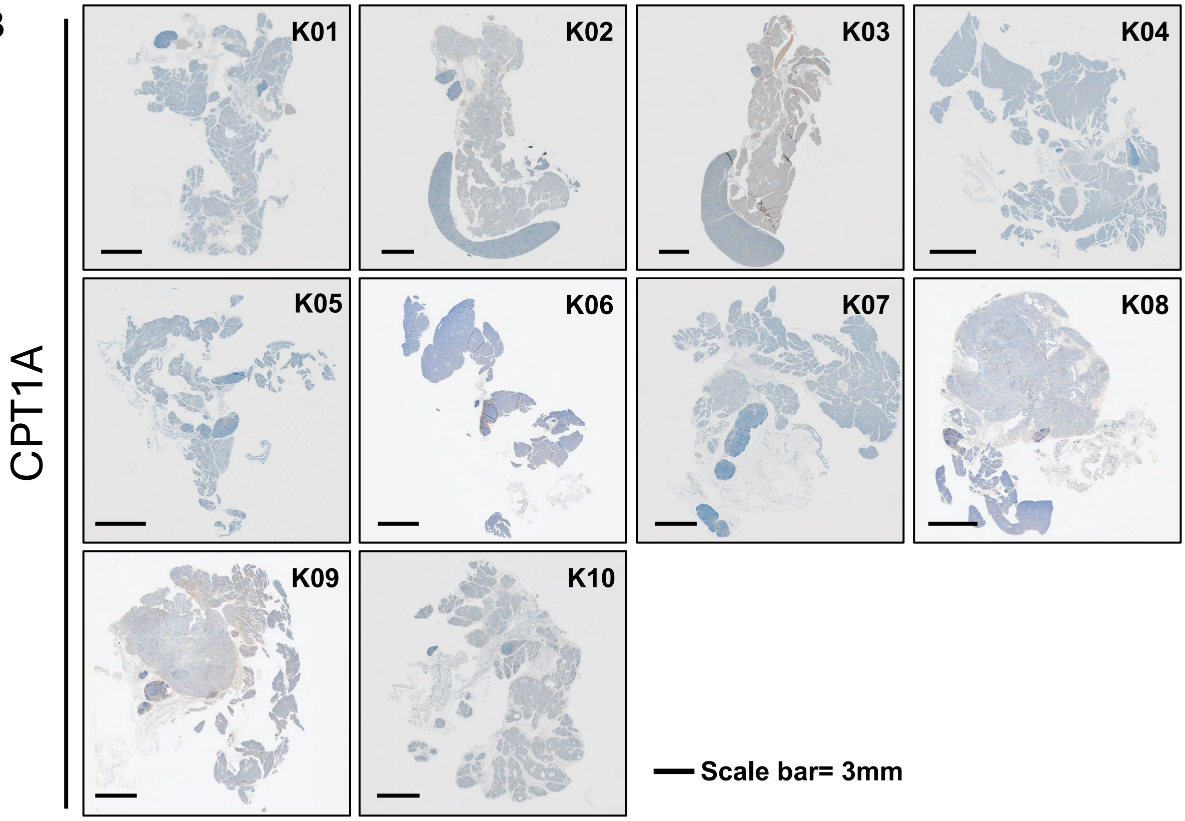
CPT1A
K01
K02
K03
K04
K05
K06
K07
K08
K09
K10
Scale bar= 3mm

C

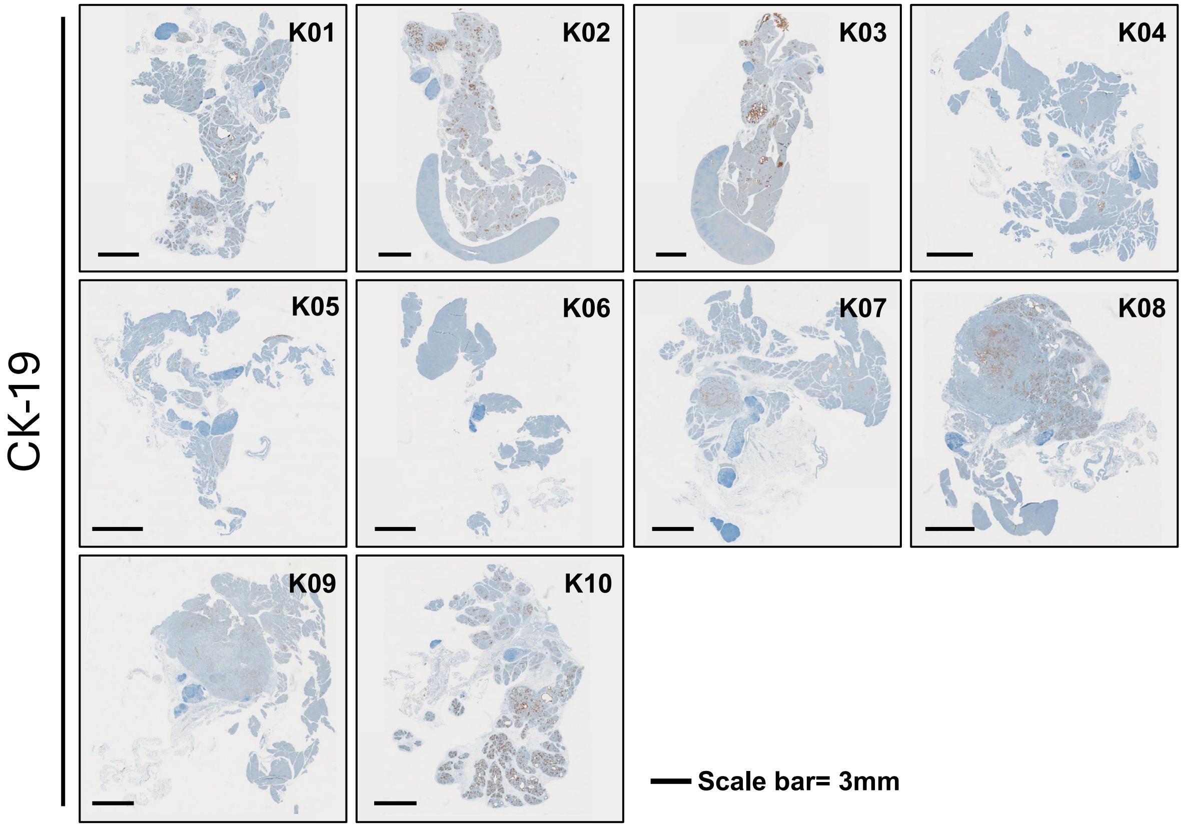
CK-19
K01
K02
K03
K04
K05
K06
K07
K08
K09
K10
Scale bar= 3mm

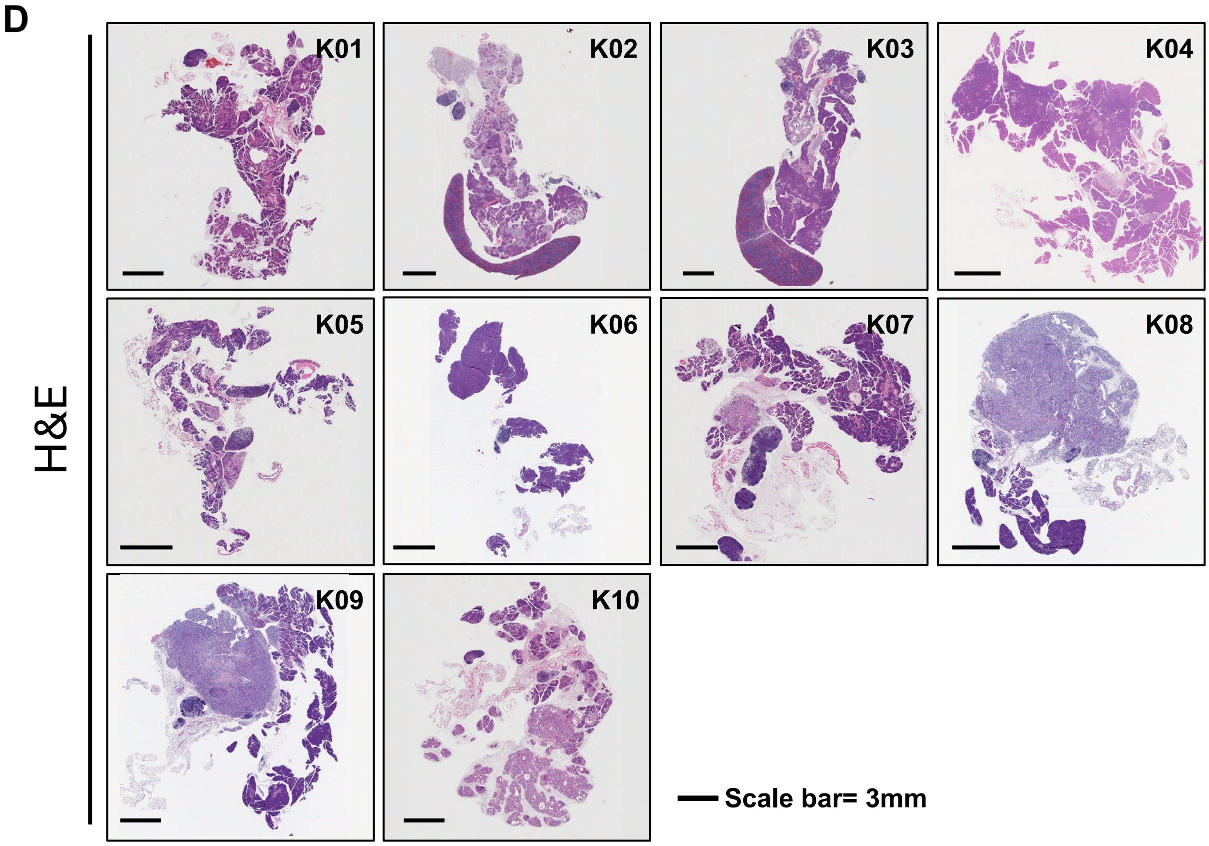

Supplementary Figure S1. CPT1A expression in PDAC TMA. (A) Pancreatic carcinoma tissue microarray (TMA) containing 60 adenocarcinoma cases and 9 normal tissue samples, with triplicate cores per case (PA2072a) were purchased from Tissue Array (Derwood, MD, US). IHC images of TMA (PA2072a) stained with anti-CPT1A (ab234111). Red boxes highlight the images shown in Figure 1*A*. (Scale bar = 2 mm). (B-D) H&E and IHC staining images of KPC mouse tissue shown in Figure 1F-H. (B) Immunohistochemistry (IHC) image highlights CPT1A expression, (C) IHC image shows CK19 expression, and (D) Hematoxylin and eosin (H&E) staining image illustrates the histological architecture within the same tissues.

Supplementary Figure S2

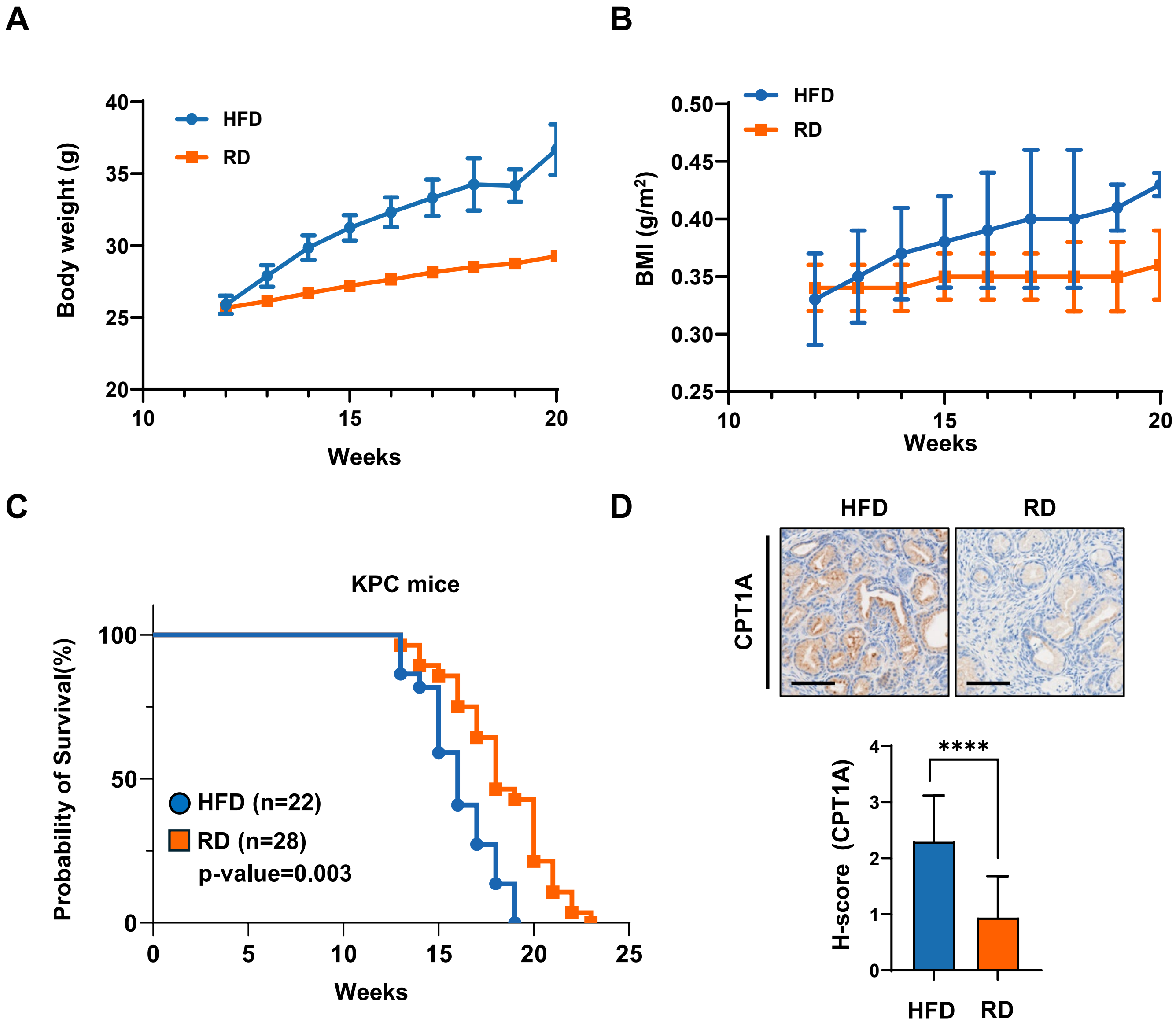

Supplementary Figure S2. HFD promotes the growth of pancreatic cancer and reduces the survival rates of xenografted mice. (A) The body weight change curve of the KPC mice fed an HFD (blue, 60% fat, n = 22) or a regular diet (orange, RD, 18% fat, n = 28). (B) BMI of KPC mice fed an HFD (blue) or an RD (orange). (C) Kaplan-Meier survival curves showed the HFD group (blue) survived for 2 weeks less than the RD group (orange). The difference in survival rates was significant (p-value=0.003). (D) IHC images of CPT1A in KPC mice with HFD (n=11) or with an RD (n=10). ****p < 0.0001.

Supplementary Figure S3

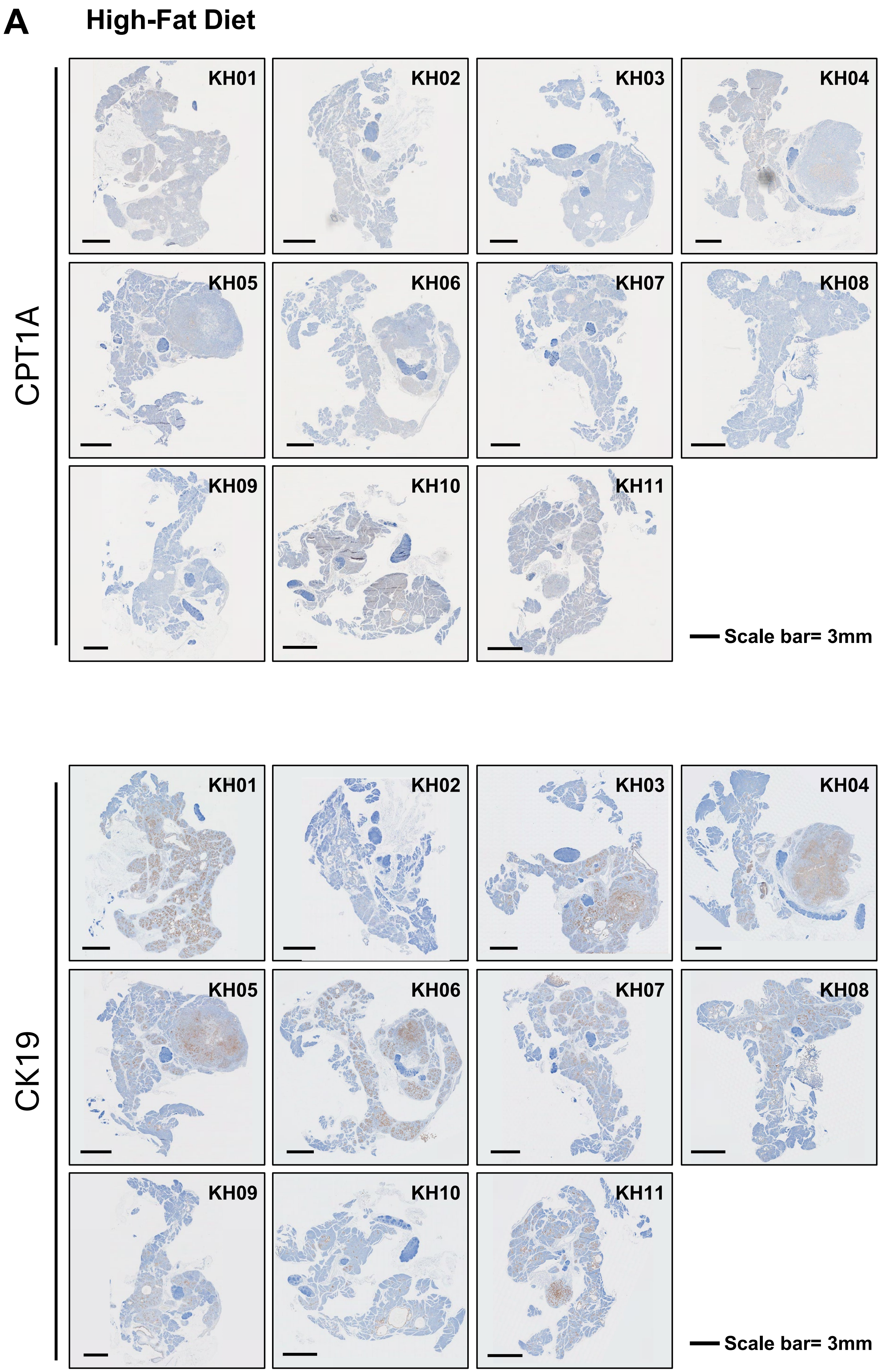

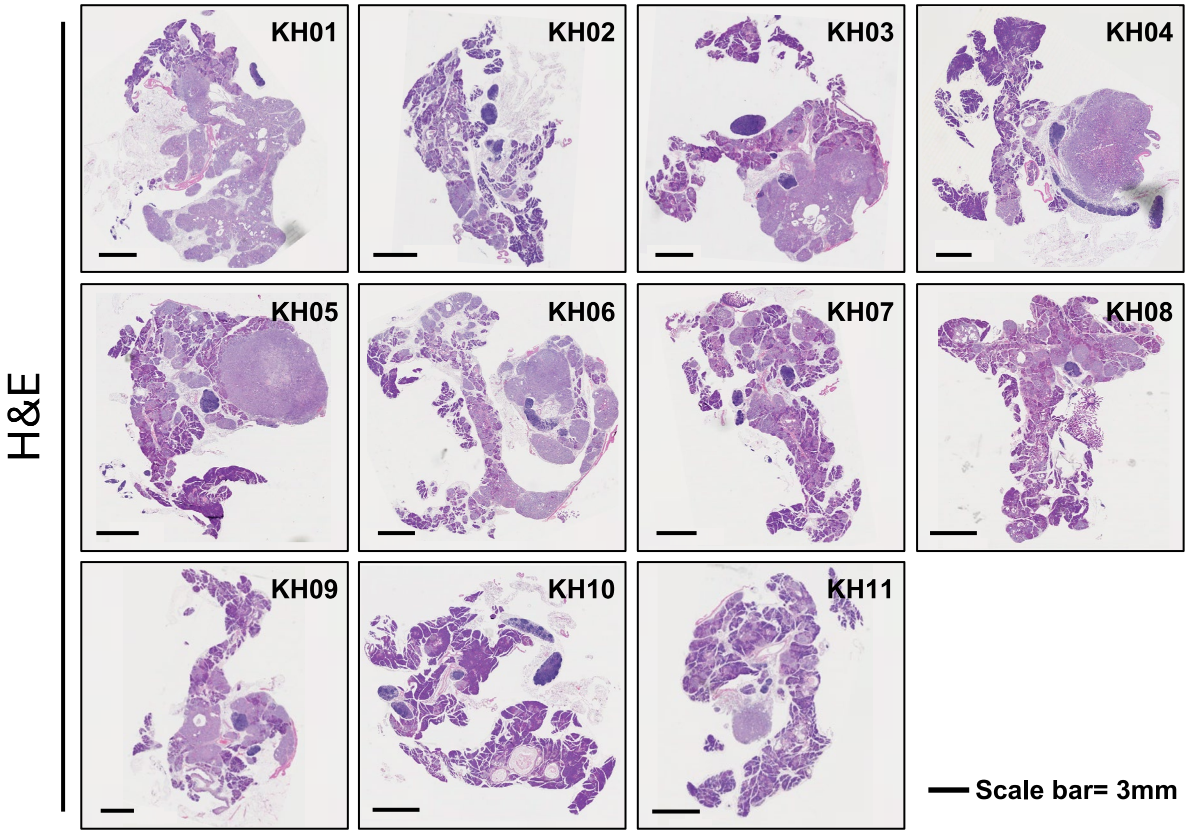
H&E
KH01
KH02
KH03
KH04
KH05
KH06
KH07
KH08
KH09
KH10
KH11
Scale bar= 3mm

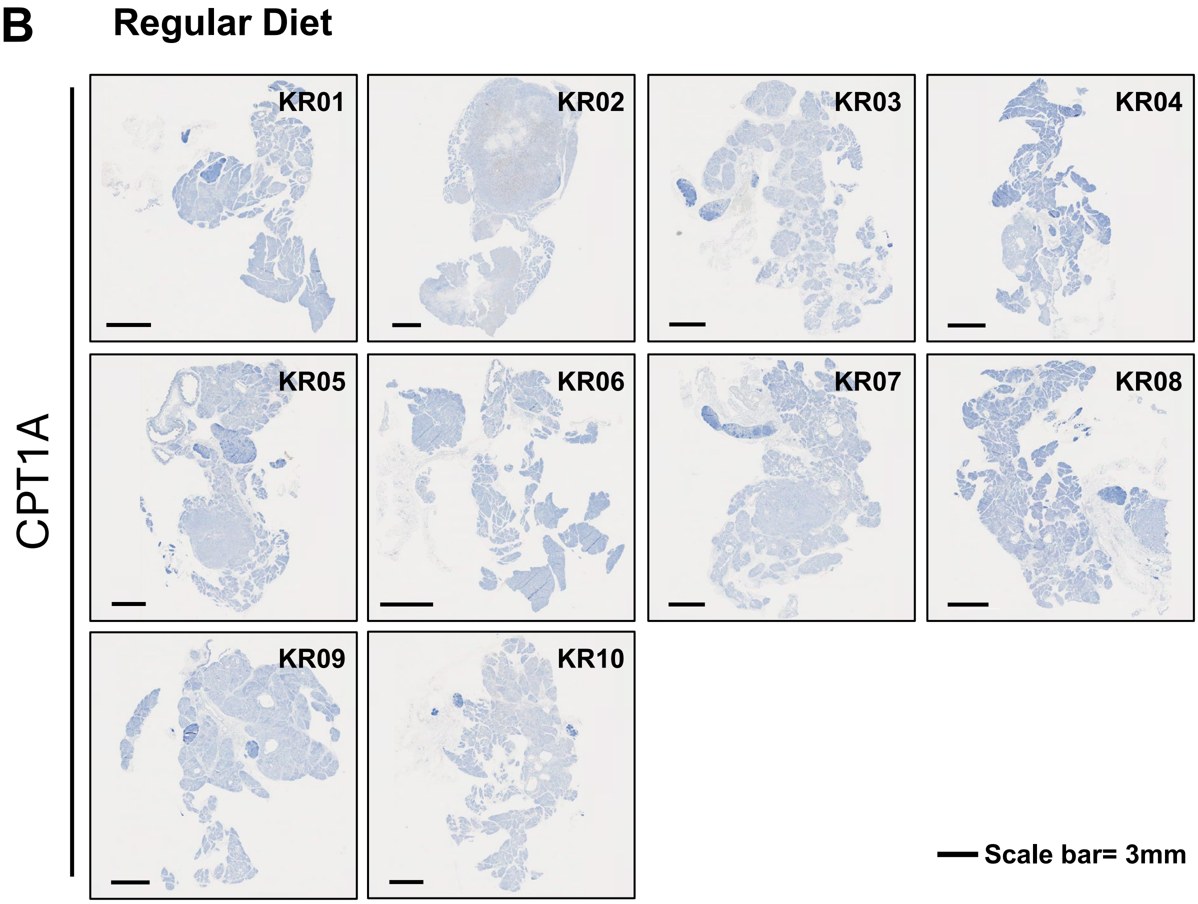
B
Regular Diet
CPT1A
KR01
KR02
KR03
KR04
KR05
KR06
KR07
KR08
KR09
KR10
Scale bar= 3mm

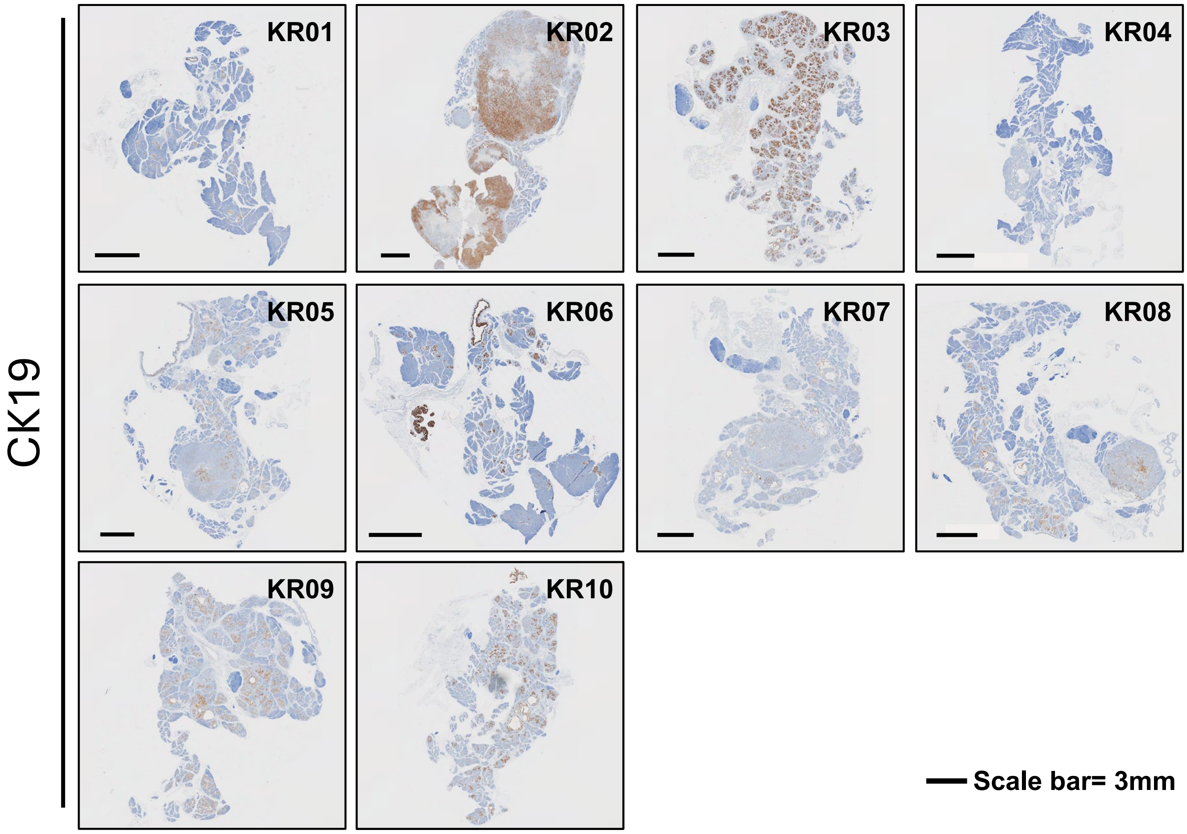
CK19
KR01
KR02
KR03
KR04
KR05
KR06
KR07
KR08
KR09
KR10
Scale bar= 3mm

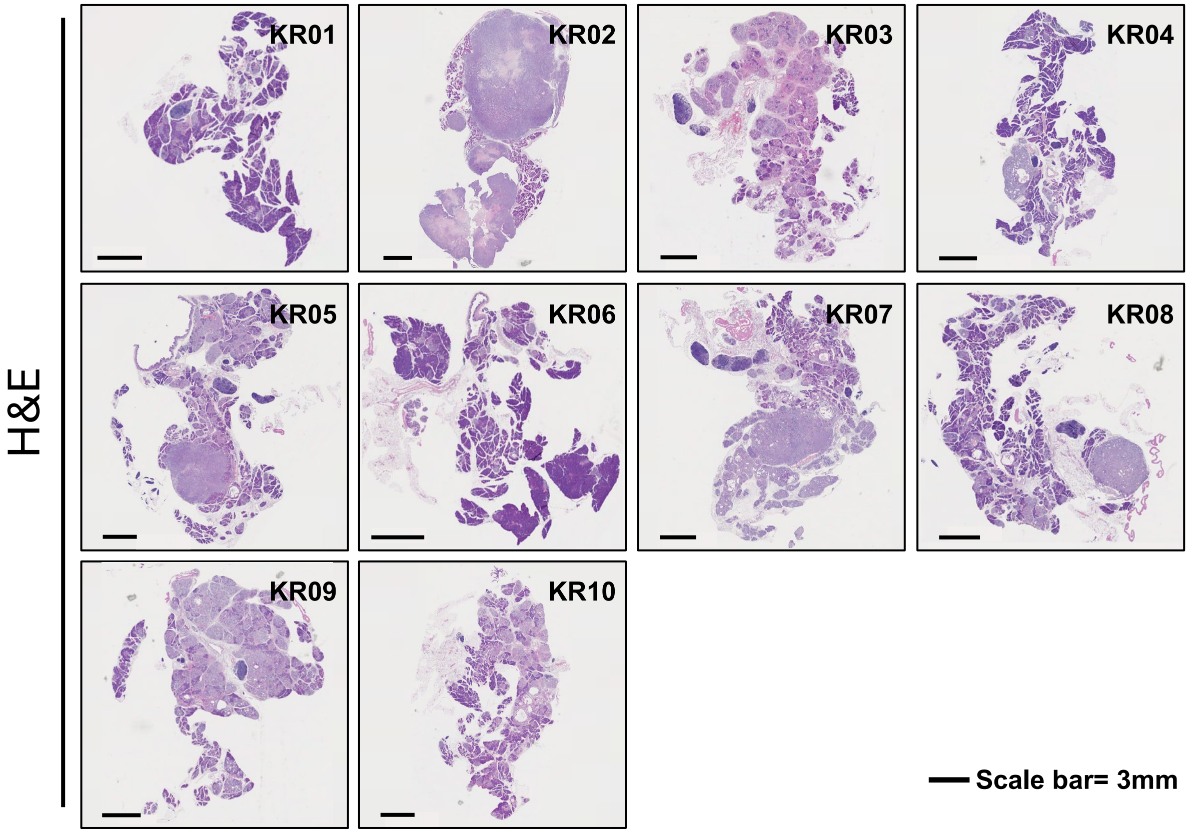

Supplementary Figure S3. Immunohistochemistry and H&E staining of the pancreas from KPC mice. (A) Fed by High-Fat Diet, (B) Fed by Regular Diet.

Supplementary Figure S4

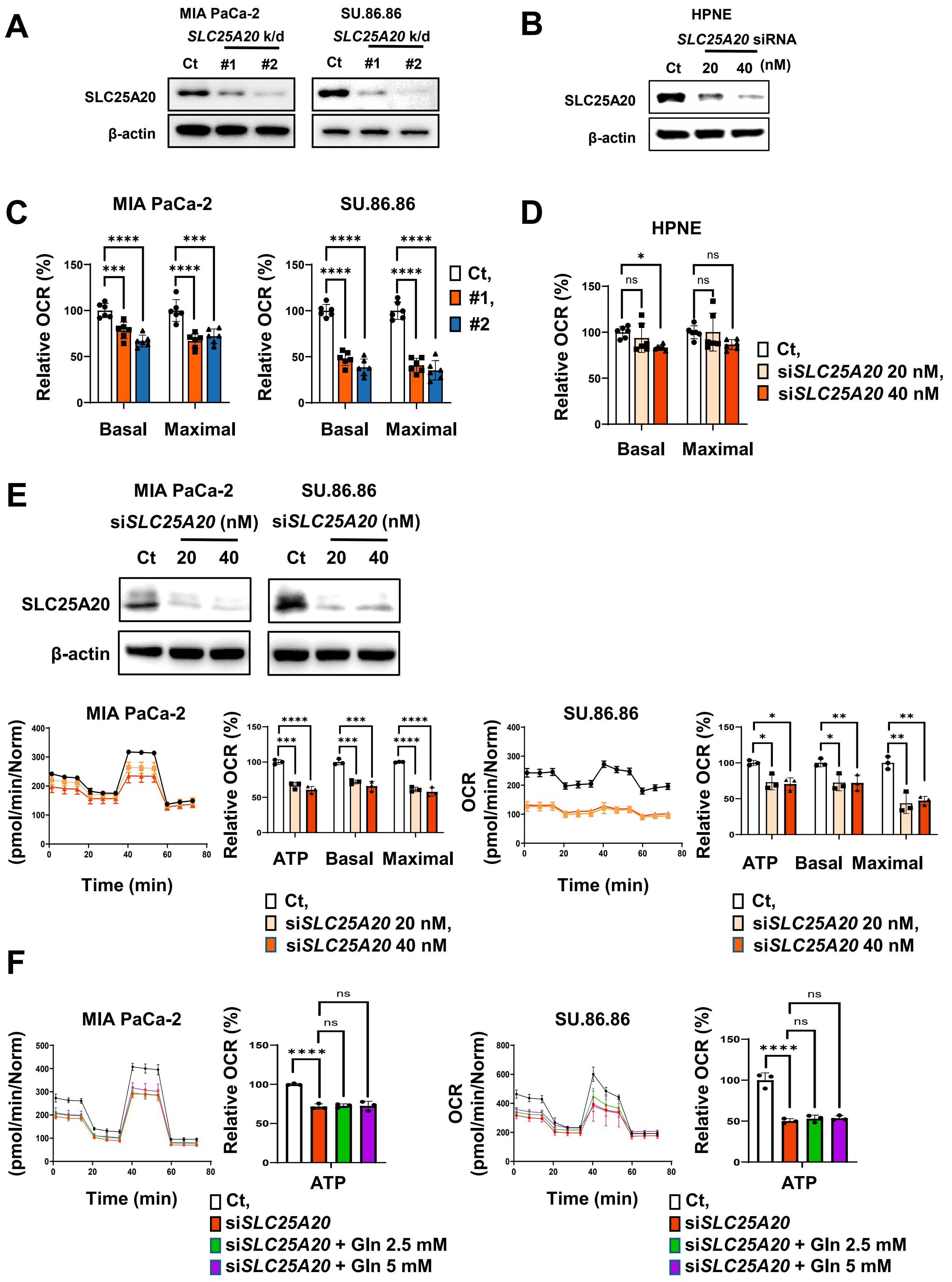

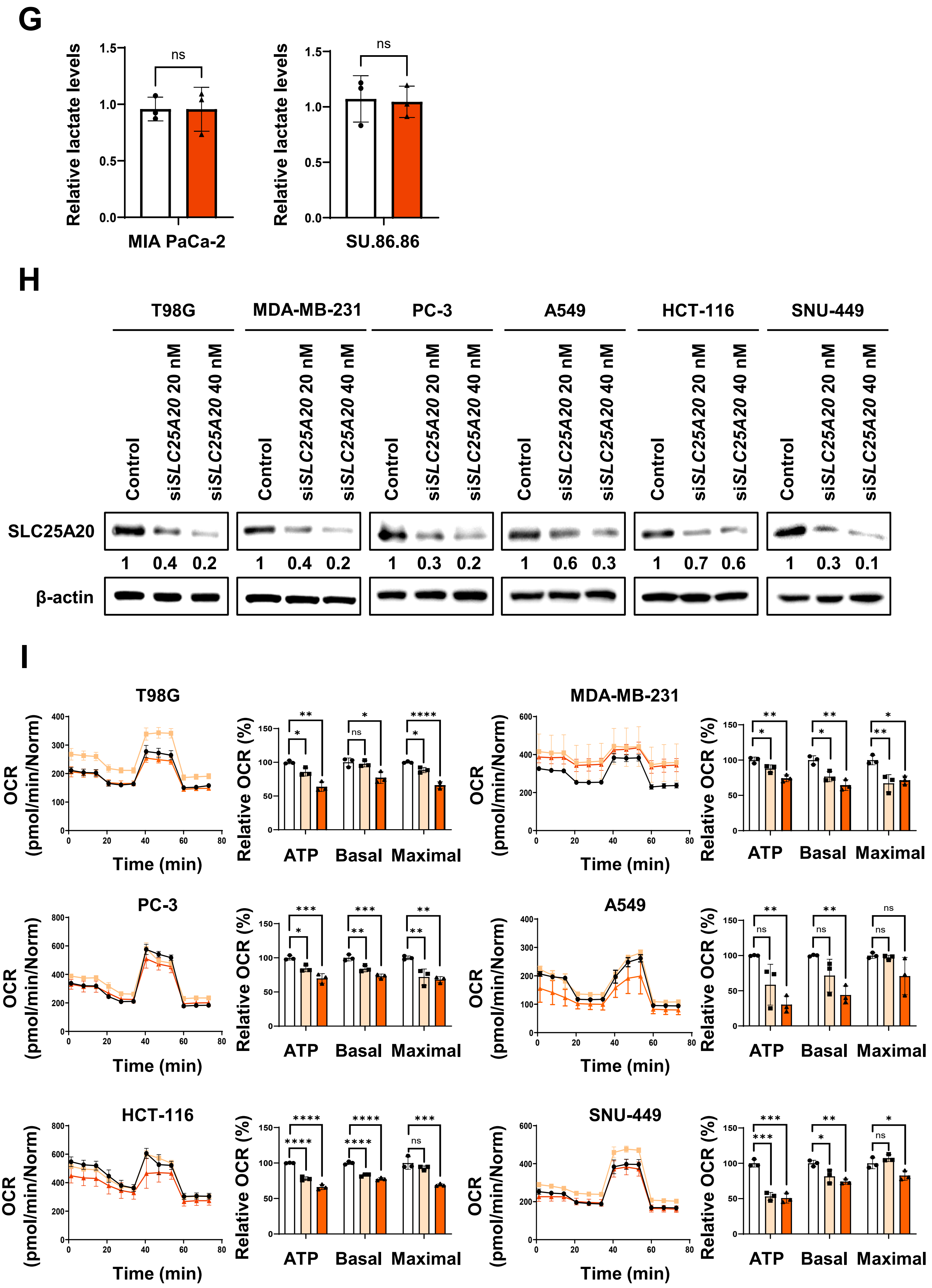
G
Relative lactate levels
ns
MIA PaCa-2
SU.86.86
H
T98G
MDA-MB-231
PC-3
A549
HCT-116
SNU-449
Control
siSLC25A20 20 nM
siSLC25A20 40 nM
SLC25A20
β-actin
1 0.4 0.2
1 0.4 0.2
1 0.3 0.2
1 0.6 0.3
1 0.7 0.6
1 0.3 0.1
I
T98G
MDA-MB-231
PC-3
A549
HCT-116
SNU-449
OCR (pmol/min/Norm)
Time (min)
Relative OCR (%)
ATP
Basal
Maximal

Supplementary Figure S4. Effect of *SLC25A20* knockdown on OCR between normal and pancreatic cancer cells. (A) Immunoblot analysis reveals a reduction in *SLC25A20* levels in MIA PaCa-2 and SU.86.86 cells in which *SLC25A20* was knocked down using two different shRNAs (#1 and #2). (B) Immunoblot analysis reveals a reduction in *SLC25A20* levels in HPNE in a dose dependent manner of shRNAs (#1). (C) OCRs were analyzed by Seahorse XFe96 analysis. All data were normalized by SRB quantification. MIA PaCa-2 and SU.86.86 with knockdown of *SLC25A20* by two different shRNAs of #1 and #2. (D) OCR of HPNE with *SLC25A20* knockdown were analyzed by Seahorse XFe96 analysis. (E) Immunoblotting analysis showed the decrease of *SLC25A20* in MIA PaCa-2, SU.86.86 with *SLC25A20* knockdown by two doses of siRNA for 48 h. OCR analysis were performed by siRNA of *SLC25A20* treatment. (F) OCRs were analyzed by Seahorse XFe 96 analyzer in control and 40 nM siRNA-mediated *SLC25A20* knockdown PDAC cells following treatment with 2.5 or 5 mM glutamine for 24 h. Glutamine supplementation did not rescue ATP depletion induced by *SLC25A20* knockdown. (G) Lactate levels were measured using a lactate assay kit in cultured cells under *SLC25A20* knockdown for 48 h. (H) Immunoblotting analysis showed the decrease of *SLC25A20* in T98G, MDA-MB-231, PC-3, A549, HCT-116 and SNU-449 with *SLC25A20* knockdown by two dose of siRNA. (I) ATP levels, basal respiration, and maximal respiration were analyzed by Seahorse XFe96 analysis. All data were normalized by SRB quantification. Data are presented as the mean ± SD from at least three experiments. $^{*}p < 0.05$, $^{**}p < 0.01$, $^{***}p < 0.001$.

Supplementary Figure S5

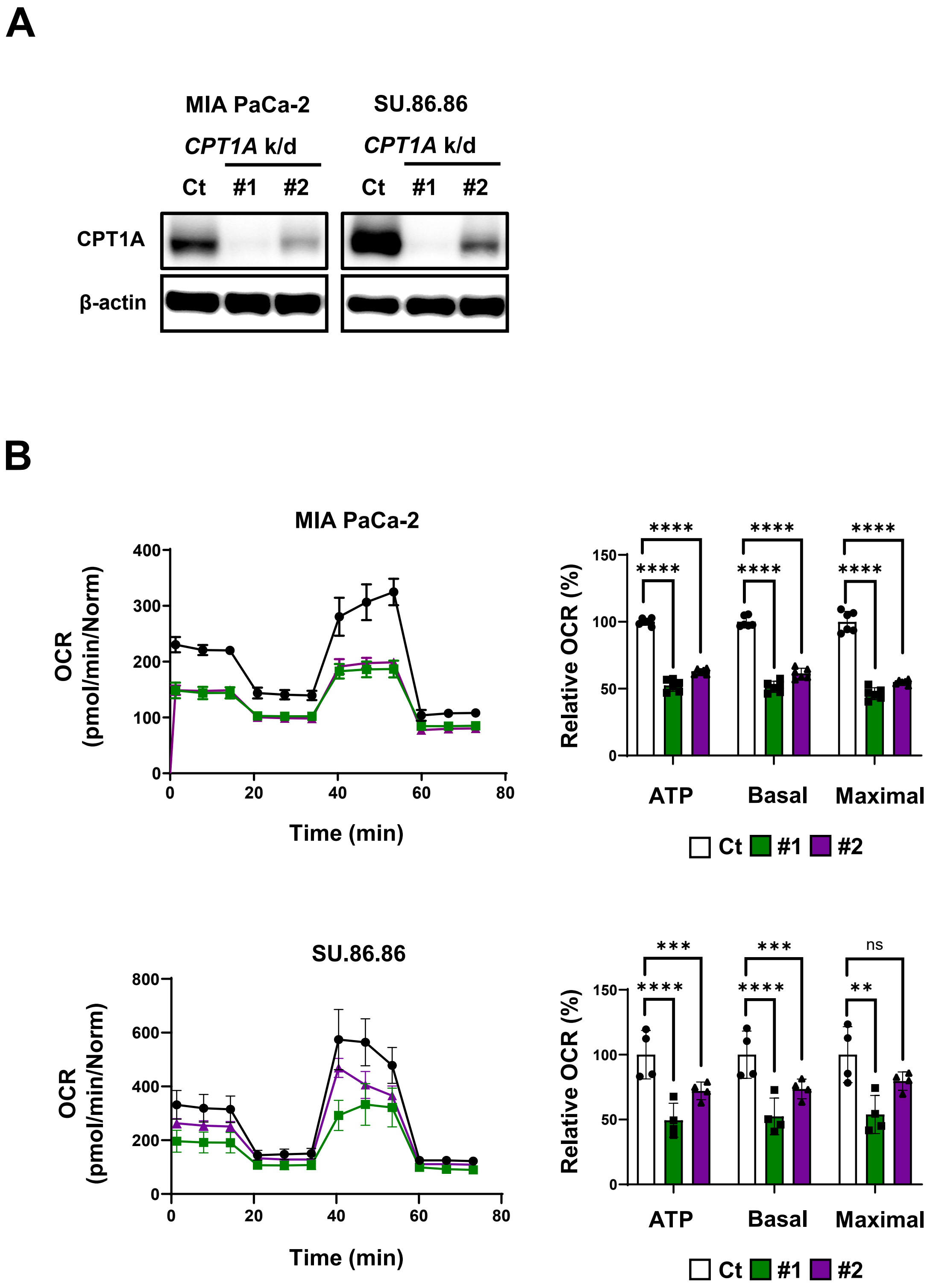

Supplementary Figure S5. Effect of *CPT1A* knockdown on ATP production in the PDAC cells. (A) Immunoblotting analysis showed the decrease of *CPT1A* in MIA PaCa-2 and SU.86.86 with *CPTIA* knockdown by two different shRNAs of #1 and #2. (B) ATP levels, basal respiration, and maximal respiration were analyzed by Seahorse XFe96 analysis in MIA PaCa-2 and SU.86.86. All data were normalized by SRB quantification.

Supplementary Figure S6

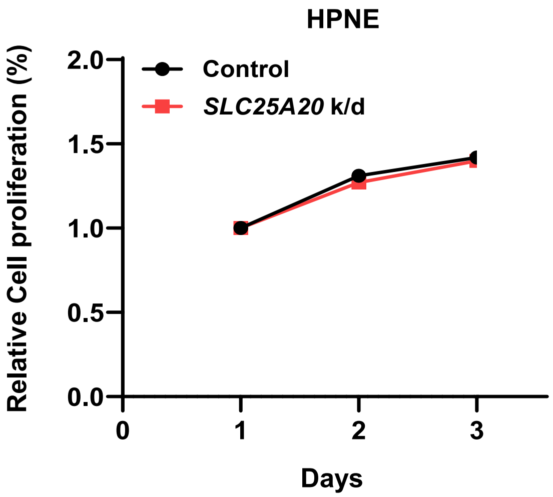

Supplementary Figure S6. Knockdown of *SLC25A20* in pancreatic normal ductal epithelial cells (HPNE) did not affect cell proliferation. HPNE cells were transfected with Scramble siRNA or *SLC25A20* siRNA for 3 days. SRB assay was performed, and the absorbance was measured at 515 nm to assess relative cell proliferation.

Supplementary Figure S7

A

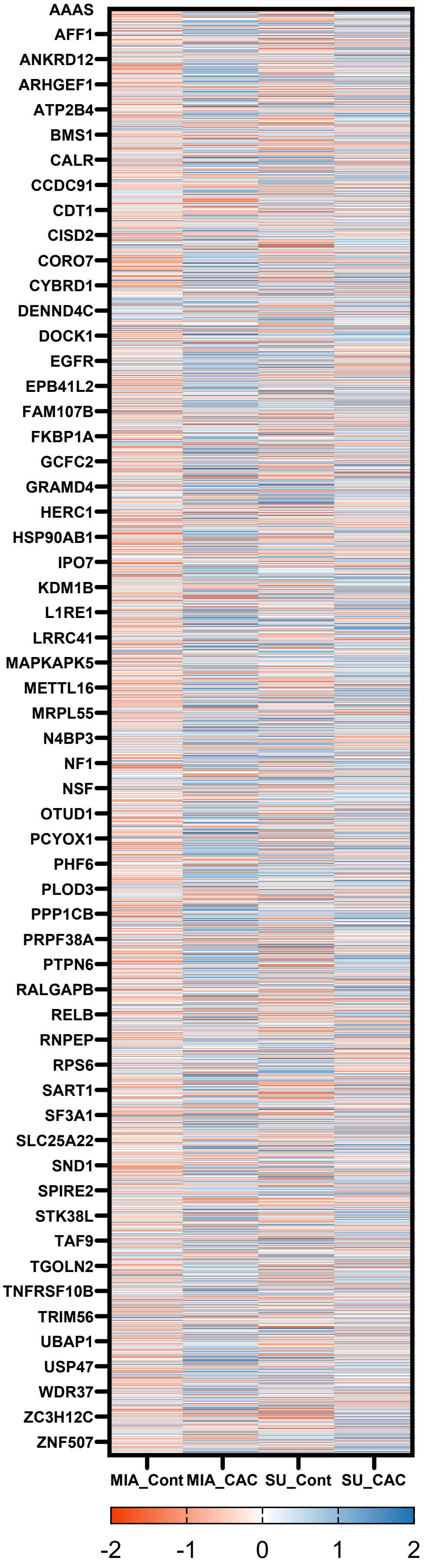

B

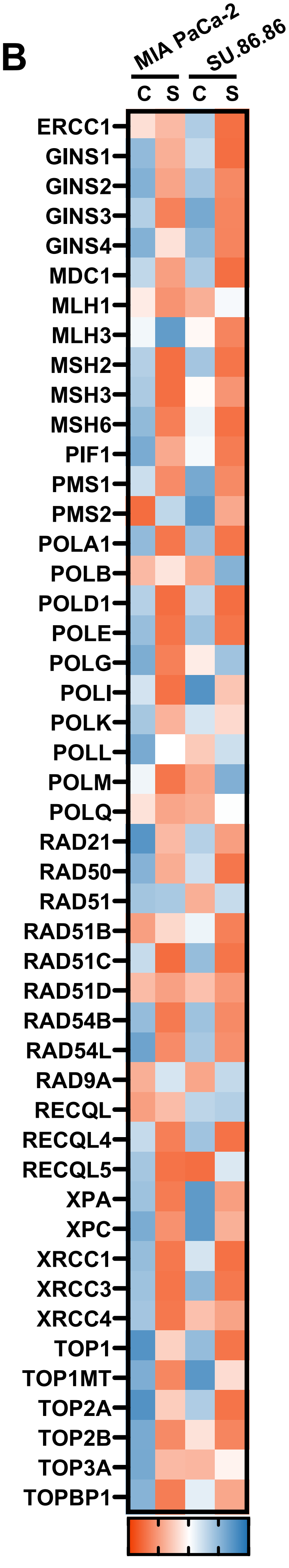

C

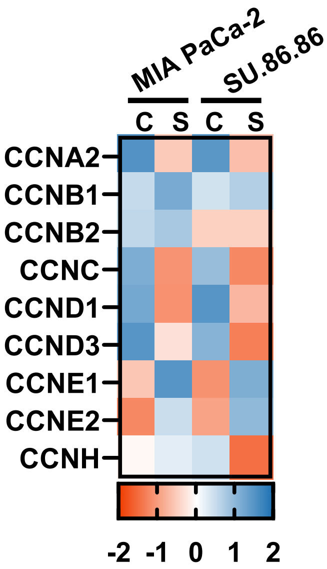

D

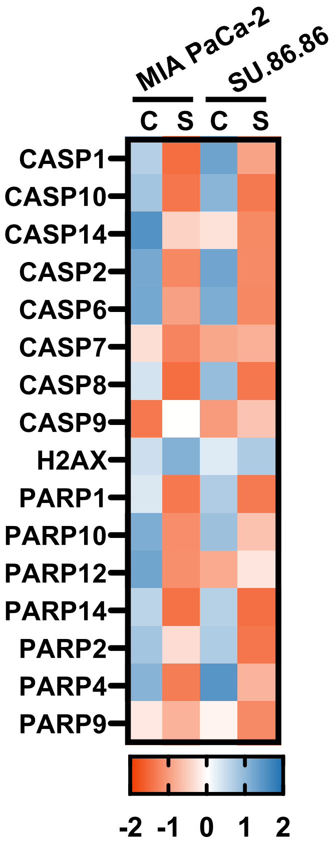

Supplementary Figure S7. Protein Phosphorylation Changes Induced by *SLC25A20* Inhibition. (A) MIA PaCa-2 and SU.86.86 cells were treated with siRNA (40 nM) for the *SLC25A20* gene for 48 h, and total protein phosphorylation was analyzed using LC-MS/MS. (B-C) After knocking down the *SLC25A20* gene in pancreatic cancer cell lines for 72 hours, proteomics analysis was performed. (B) DNA damage repair-related proteins (C) cell cycle checkpoint proteins (D) apoptotic cell death related proteins. All graph represent the z-score.

Supplementary Figure S8

A. Figure 5D

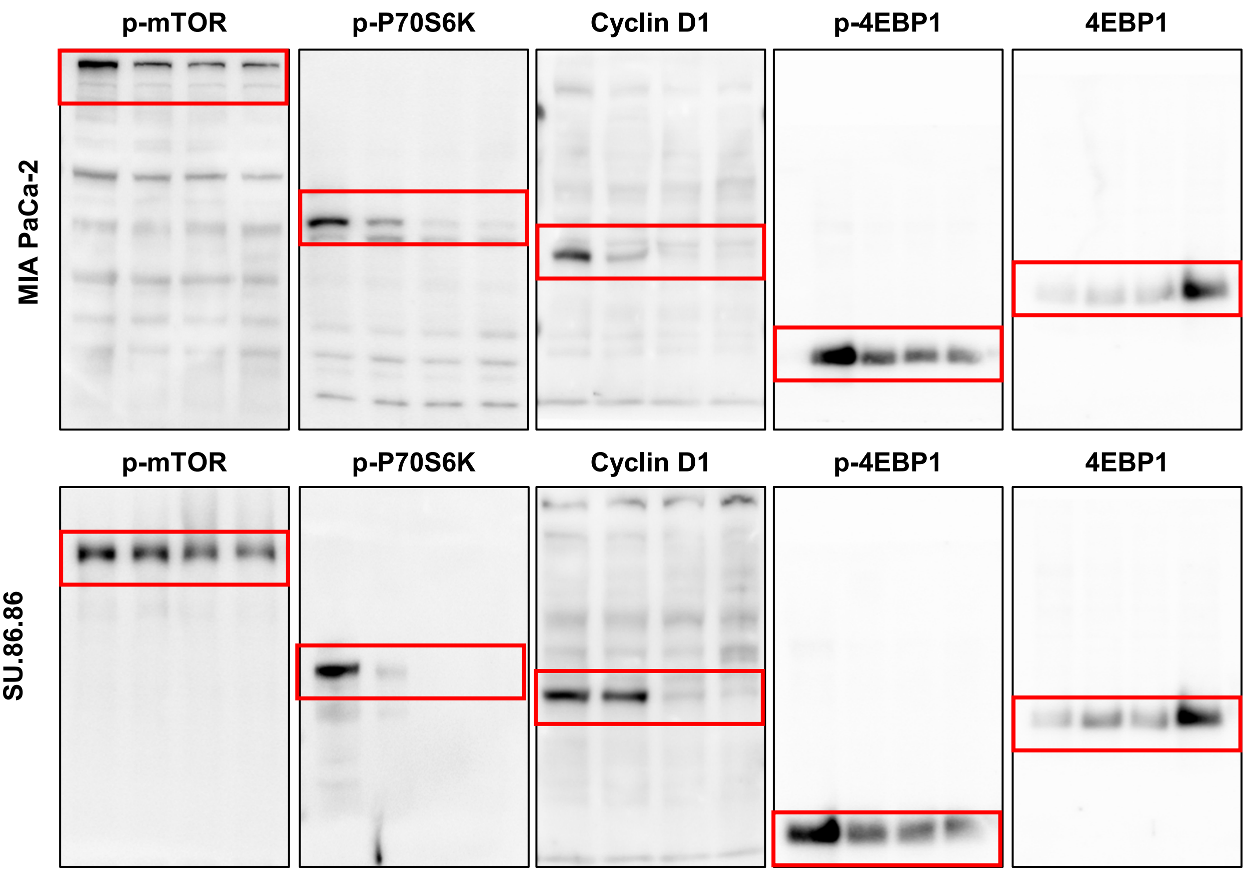

B. Figure 5E

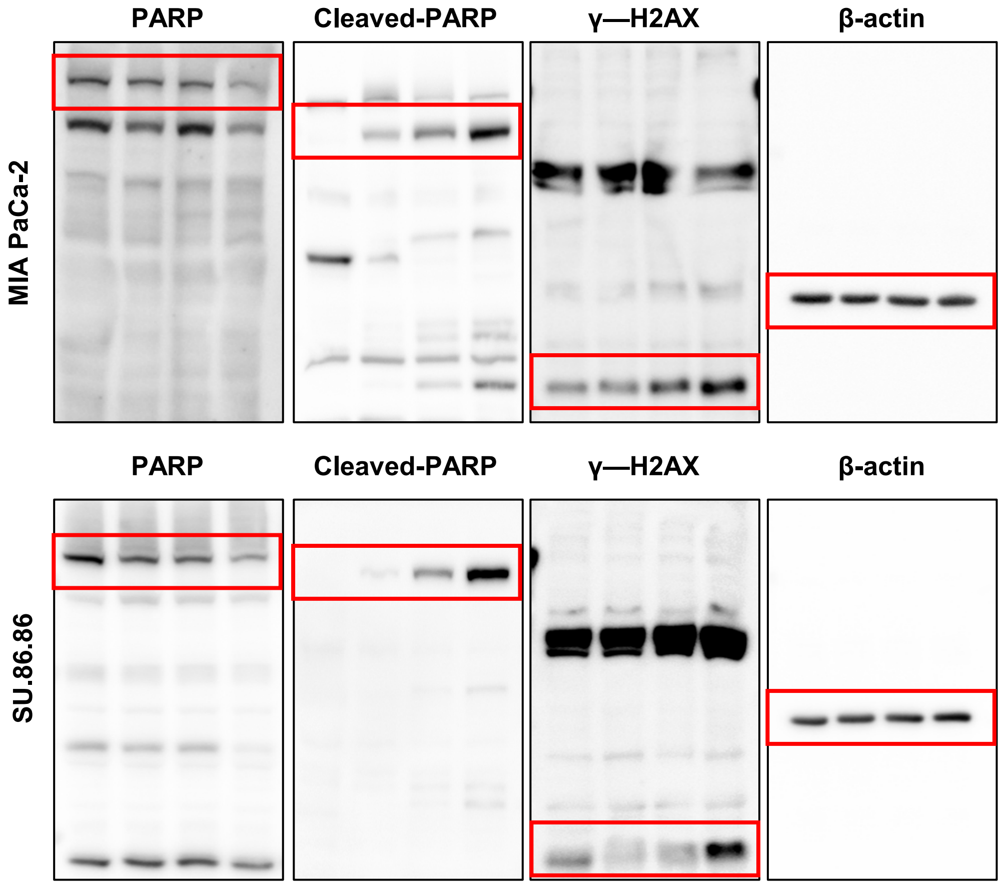

Supplementary Figure S8. *SLC25A20* knockdown reduces cell proliferation and increases cell death. Time-dependent treatment with *SLC25A20* siRNA was performed in MIA PaCa-2 and SU.86.86 cells. (A) The key markers of cell proliferation, mTOR, P70S6K, and 4EBP1, were analyzed by immunoblotting. (B) In the same samples, cell death markers such as PARP, cleaved PARP, and γ-H2AX were examined by immunoblotting.

Supplementary Figure S9

A

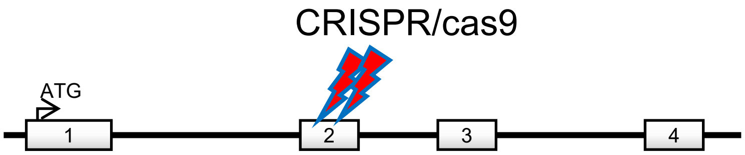

- WT-exon2:
- 5′-GTCCGACTGC AGACACAACC ACCAAGTTTG TCTGGACAGC CACCTATGTA CTCTGGGACC TTGGACTGTT TCCGGAAGAC TCTTATGAGA GAG

- mut-exon2:
- 5′- GTCCGACTGC AGACACAACC ACCAAGTTTG TCTGGACAGC CAC------- CTCTGGGACC TTGGACTGTT TCCGGAAGAC TCTTATGAGA GAG

* Protein translation (out-of-frame)
Wt-exon2: ~~**GQPP**MYSGTLDCFRKTLMRE~~
mut(indel): ~~***GQPP****LGPWTVSGRLL***

B

- Genotype PCR Primer

Slc25a20_For: TG GAT CGA AGG ATG AGG AGC AG
Slc25a20_Rev: AT CTC ACA GCT ACC TTC TTC AC

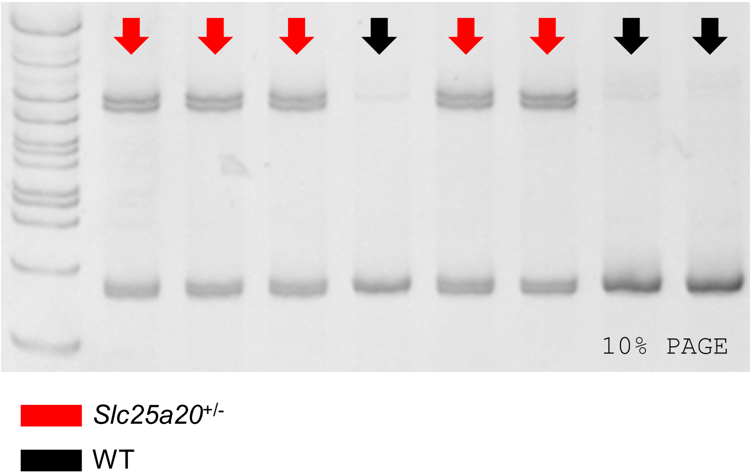

Supplementary Figure S9. Generation of *Slc25a20*-null mouse carnitine acylcarnitine carrier (7nt deletion). (A) Schematic of *Slc25a20* knockout mouse generation. The *SLC25A20* gene was targeted for knockout using CRISPR/Cas9. (B) Genotyping PCR primers and 10% polyacrylamide gel electrophoresis of genotyping PCR products. Representative PAGE gel showed that the red arrow indicates the band corresponding to the *Slc25a20* knockout allele, and the black arrow indicates the wild-type allele.

Supplementary Figure S10

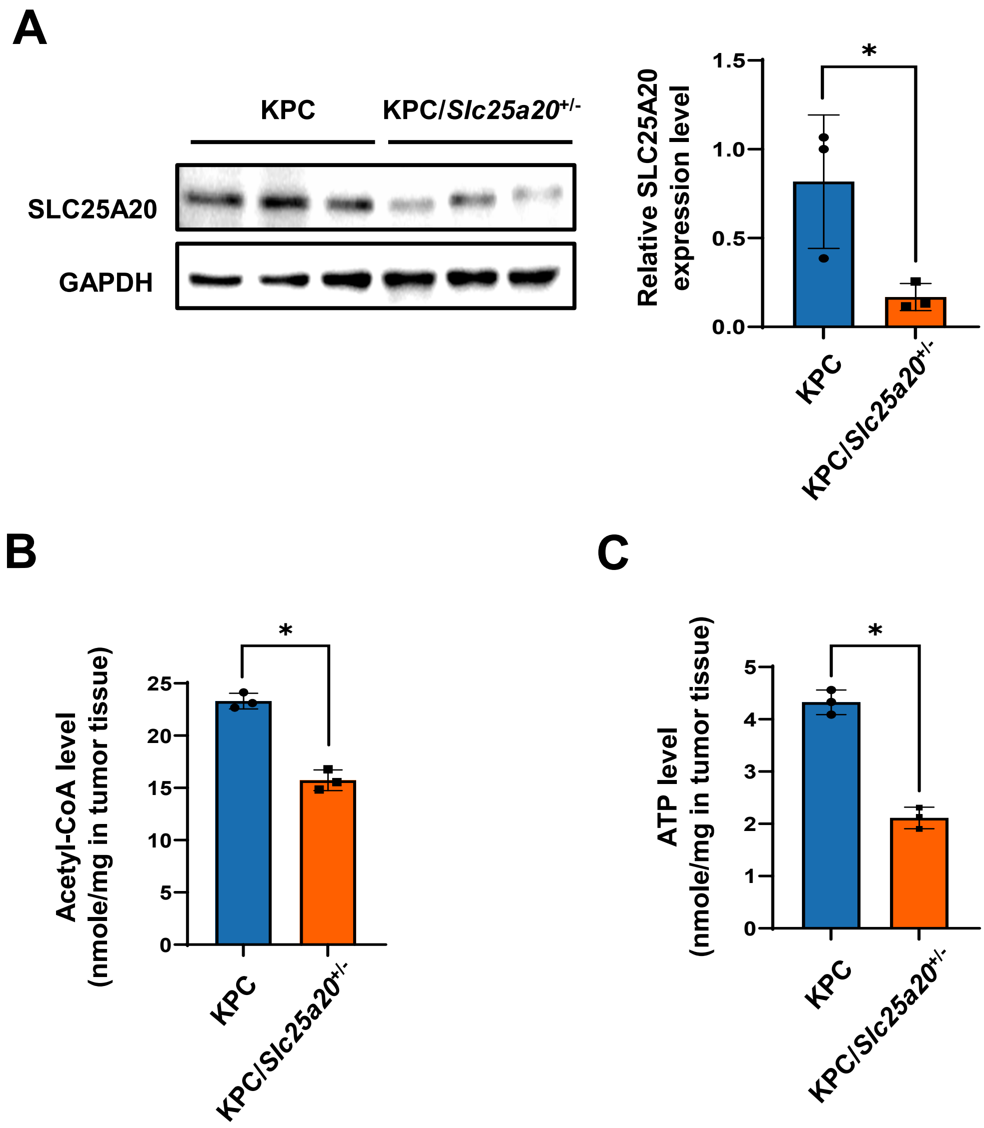

Supplementary Figure S10. *SLC25A20* knockdown in KPC mice reduces FAO metabolism. The final metabolite of FAO is acetyl-CoA, which is synthesized into ATP via TCA-OxPhos. Therefore, the decrease in acetyl-CoA and ATP in KPC/*Slc25a20*$^{+/-}$ mice indicates a decrease in FAO. (A) Immunoblot analysis revealed a reduction in SLC25A20 levels in pancreatic tumor tissues of KPC (n=3, age (weeks): 19, 12, 10) and KPC*/Slc25a20*$^{+/-}$ (n=3, age (weeks): 18, 16, 15) mouse. (B) Acetyl-CoA levels and (C) ATP levels in pancreatic tumor tissues. Data are presented as the mean ± SD *p < 0.05.

Supplementary Figure S11

A KPC/*Slc25a20*$^{+/-}$ mice

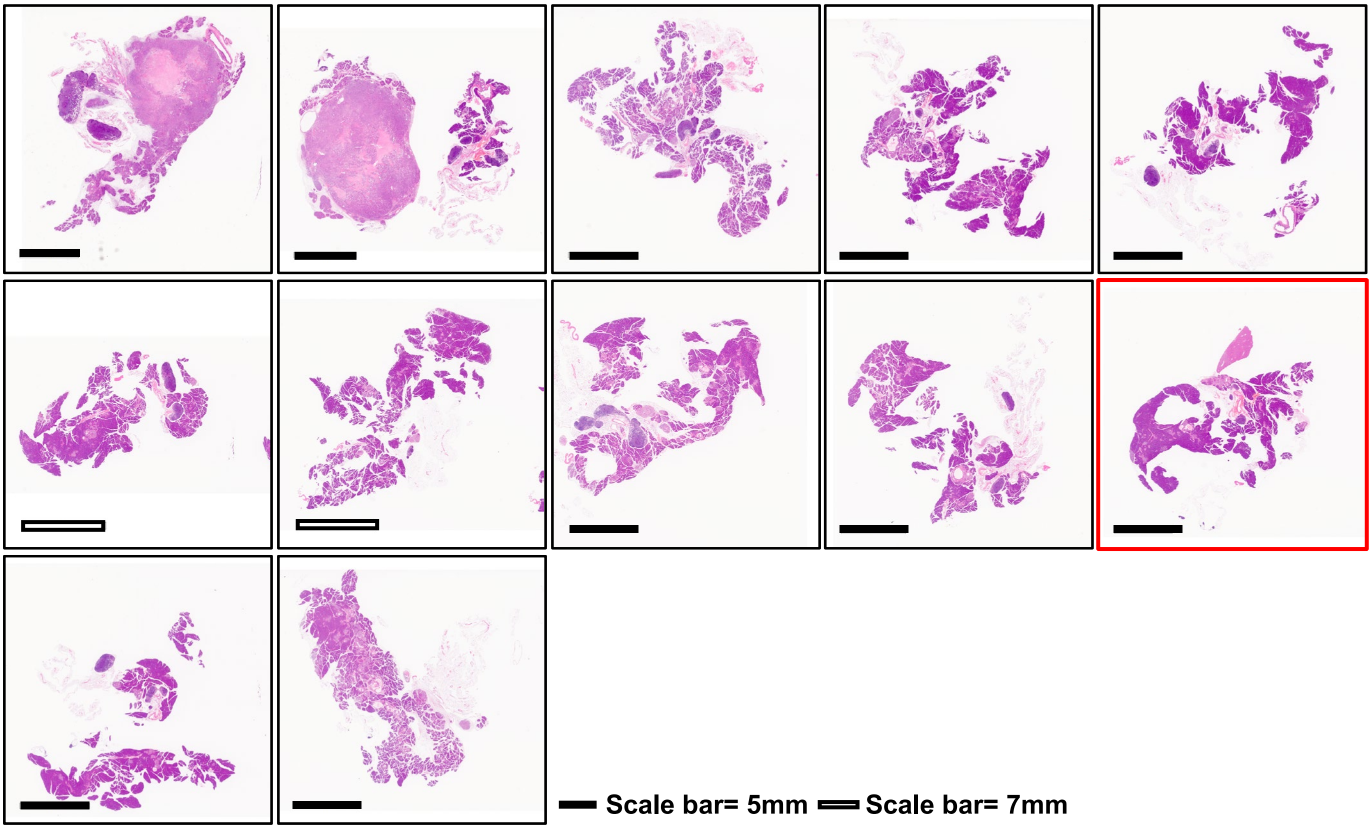

B KPC mice

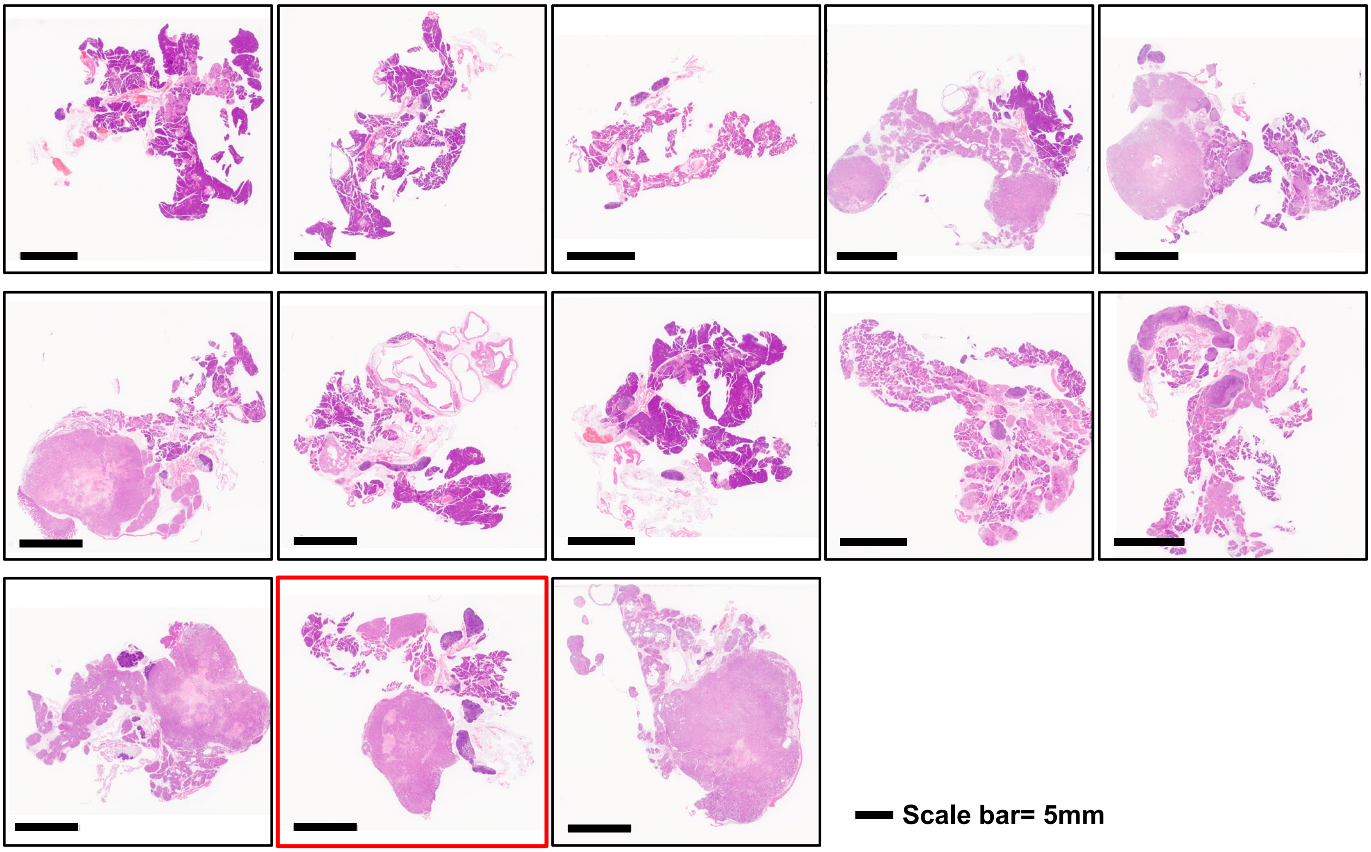

C

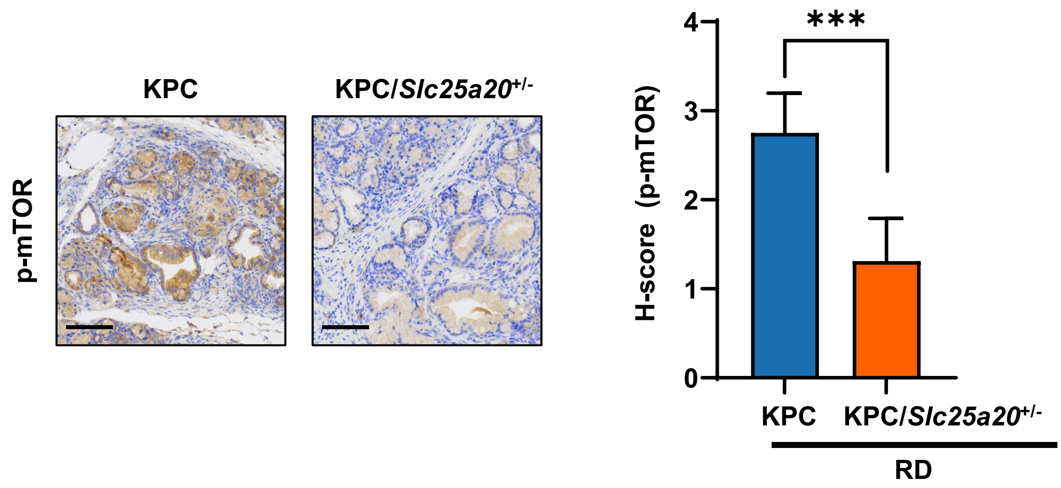

KPC
KPC/Slc25a20+/-
p-mTOR
***
4
3
2
1
0
H-score (p-mTOR)
KPC
KPC/Slc25a20+/-
RD

D

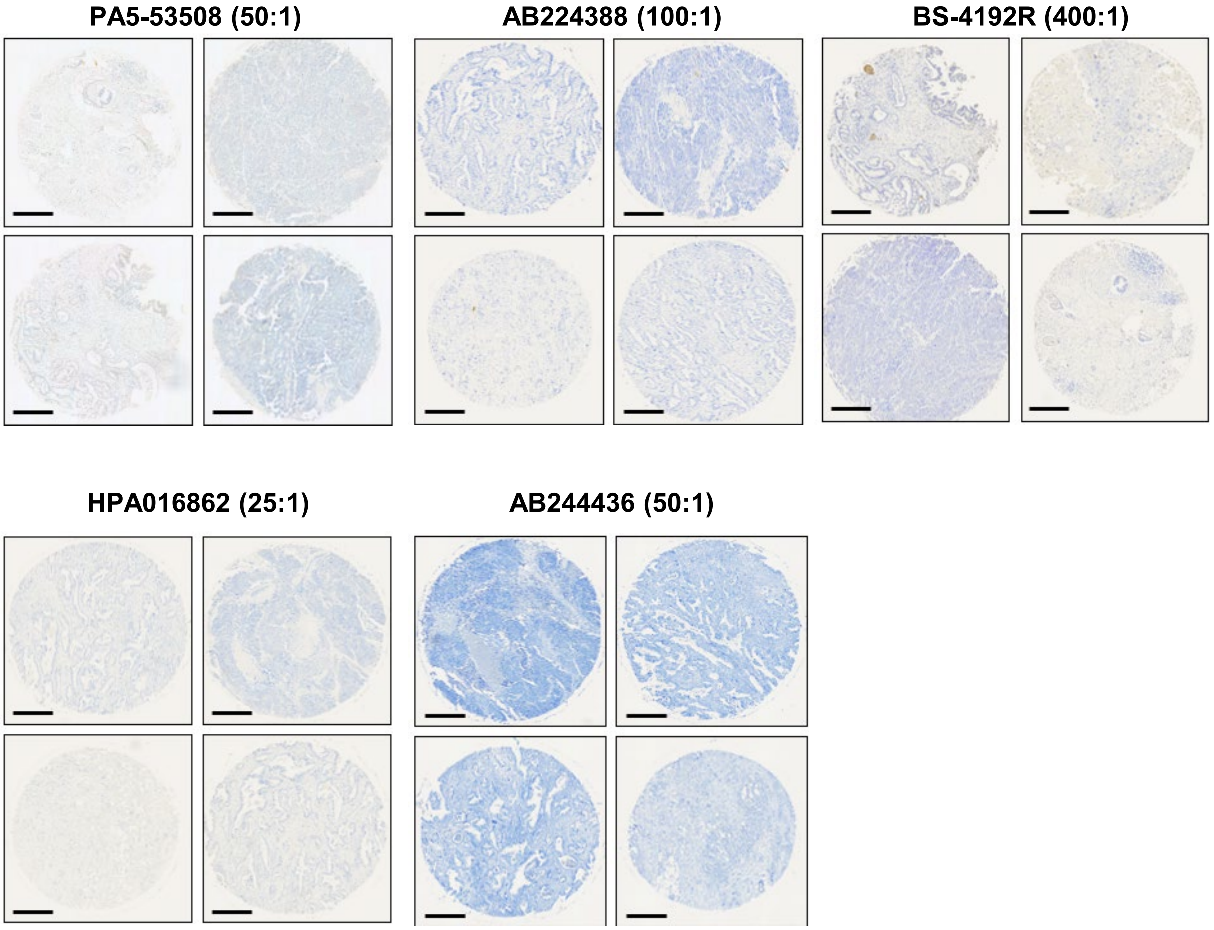

PA5-53508 (50:1)
AB224388 (100:1)
BS-4192R (400:1)
HPA016862 (25:1)
AB244436 (50:1)

Supplementary Figure S11. H&E staining images of KPC and KPC/*Slc25a20*$^{+/-}$ mice shown in Figure 7. (A) Pancreas tissue from KPC/*Slc25a20*$^{+/-}$ mice, (B) Pancreas tissue from KPC mice. (C) Immunohistochemical (IHC) analysis of p-mTOR expression in 20-week-old KPC/Slc25a20+/- and KPC pancreatic cancer tissues. (Scale bar: 100 μm) The median H-score for p-mTOR in KPC and KPC/Slc25a20+/- was 2.75 and 1.31, respectively. (D) Immunohistochemical (IHC) analysis of SLC25A20 expression in TMA (T142C) tissue using various anti-SLC25A20 antibodies. IHC staining was performed using the following anti-SLC25A20 antibodies: PA5-53508 (1:50 dilution, Invitrogen), AB224388 (1:100 dilution, Abcam), BS-4192R (1:400 dilution, Bioss Antibodies), HPA016862 (1:25 dilution, Atlas Antibodies), and AB244436 (1:50 dilution, Abcam). No specific staining for SLC25A20 was observed in the TMA (T142C) tissue sections with any of the antibodies tested. (Scale bar: 400 μm)
